# Low-density lipoprotein receptor promotes crosstalk between cell stemness and tumor immune microenvironment in breast cancer: a large data-based multi-omics study

**DOI:** 10.1186/s12967-023-04699-y

**Published:** 2023-11-30

**Authors:** Qihang Yuan, Xiaona Lu, Hui Guo, Jiaao Sun, Mengying Yang, Quentin Liu, Mengying Tong

**Affiliations:** 1 Department of General Surgery, First Affiliated Hospital of Dalian Medical University, Dalian, China; 2 Institute of Cancer Stem Cell, Cancer Center, Dalian Medical University, Dalian, China; 3 Department of Urology, First Affiliated Hospital of Dalian Medical University, Dalian, China; 4 State Key Laboratory of Oncology in South China, Cancer Center, Sun Yat-sen University, Guangzhou, China; 5 Department of Ultrasound, First Affiliated Hospital of Dalian Medical University, Dalian, China

**Keywords:** Breast cancer, Cell stemness, Tumor immune microenvironment, LDLR, Multi-omics study

## Abstract

**Background:**

Tumor cells with stemness in breast cancer might facilitate the immune microenvironment’s suppression process and led to anti-tumor immune effects. The primary objective of this study was to identify potential targets to disrupt the communication between cancer cell stemness and the immune microenvironment.

**Methods:**

In this study, we initially isolated tumor cells with varying degrees of stemness using a spheroid formation assay. Subsequently, we employed RNA-seq and proteomic analyses to identify genes associated with stemness through gene trend analysis. These stemness-related genes were then subjected to pan-cancer analysis to elucidate their functional roles in a broader spectrum of cancer types. RNA-seq data of 3132 patients with breast cancer with clinical data were obtained from public databases. Using the identified stemness genes, we constructed two distinct stemness subtypes, denoted as C1 and C2. We subsequently conducted a comprehensive analysis of the differences between these subtypes using pathway enrichment methodology and immune infiltration algorithms. Furthermore, we identified key immune-related stemness genes by employing lasso regression analysis and a Cox survival regression model. We conducted in vitro experiments to ascertain the regulatory impact of the key gene on cell stemness. Additionally, we utilized immune infiltration analysis and pan-cancer analysis to delineate the functions attributed to this key gene. Lastly, single-cell RNA sequencing (scRNA-seq) was employed to conduct a more comprehensive examination of the key gene’s role within the microenvironment.

**Results:**

In our study, we initially identified a set of 65 stemness-related genes in breast cancer cells displaying varying stemness capabilities. Subsequently, through survival analysis, we pinpointed 41 of these stemness genes that held prognostic significance. We observed that the C2 subtype exhibited a higher stemness capacity compared to the C1 subtype and displayed a more aggressive malignancy profile. Further analysis using Lasso-Cox algorithm identified LDLR as a pivotal immune-related stemness gene. It became evident that LDLR played a crucial role in shaping the immune microenvironment. In vitro experiments demonstrated that LDLR regulated the cell stemness of breast cancer. Immune infiltration analysis and pan-cancer analysis determined that LDLR inhibited the proliferation of immune cells and might promote tumor cell progression. Lastly, in our scRNA-seq analysis, we discovered that LDLR exhibited associations with stemness marker genes within breast cancer tissues. Moreover, LDLR demonstrated higher expression levels in tumor cells compared to immune cells, further emphasizing its relevance in the context of breast cancer.

**Conclusion:**

LDLR is an important immune stemness gene that regulates cell stemness and enhances the crosstalk between breast cancer cancer cell stemness and tumor immune microenvironment.

**Supplementary Information:**

The online version contains supplementary material available at 10.1186/s12967-023-04699-y.

## Background

Breast cancer ranks among the most prevalent malignancies affecting women and typically originates in the glandular epithelial tissue of the breast. It accounts for 7–10% of the overall cancer incidence. The occurrence of breast cancer often correlates with a patient’s genetic predisposition, with a higher incidence observed in women between the ages of 40 and 60, typically around the time of menopause. However, due to escalating societal pressures and evolving dietary habits, the onset of breast cancer is occurring at increasingly younger ages. Currently, the primary clinical treatments for breast cancer continue to be surgery, radiotherapy, and chemotherapy, despite their associated adverse effects. In recent years, advancements in targeted therapy, endocrine therapy, and immunotherapy have diversified breast cancer treatment approaches. Nevertheless, breast cancer still presents a significant challenge due to its high incidence and recurrence rates.

Tumor cell stemness, as defined in our study, refers to the capability of a small subset of cells within the tumor tissue to undergo differentiation and give rise to the entire tumor. Tumor tissues are malignant tissues with unlimited proliferative potential, in which there are more tumor stem cells. Meanwhile, less cells in tumor tissues also have the function of tumor stem cells, which is the stemness characteristic of tumor cells. The augmentation of tumor cell stemness stands as a primary contributor to unfavorable tumor prognoses and the recurrence of tumors. Investigating targets for inhibiting tumor stem cells and diminishing tumor cell stemness remains a crucial and active research focus.

The tumor immune microenvironment (TIME) encompasses the microenvironment surrounding tumor cells, comprising nearby blood vessels, immune cells, fibroblasts, bone marrow-derived inflammatory cells, various signaling molecules, and the extracellular matrix. Tumors and their surroundings engage in ongoing interactions. Tumors have the capacity to shape their microenvironment by releasing cell signaling molecules, and conversely, immune cells within the microenvironment can impact cancer cell growth and development, resulting in intricate crosstalk within this milieu. Tumor cells exhibiting stemness characteristics engage in multiple crosstalk interactions with components of the immune microenvironment. These interactions enable these tumors to evade the immune response, ultimately resulting in the onset of metastasis and tumor recurrence. The interaction between tumor cell stemness and TIME exists in numerous types of tumors, and in breast [1], lung [2], esophageal [3], gastric [4, 5], colon [6], head and neck squamous cell carcinoma [7], and various pediatric tumors [8]. There exists a reciprocal relationship wherein cells within the immune microenvironment can augment tumor stemness, and conversely, cells possessing tumor stemness can impede the functionality of immune cells. This dual interaction ultimately fosters tumor metastasis and recurrence. In lung adenocarcinoma, the immune stemness genes Interleukin-6 (IL-6), Formyl peptide receptor 2 (FPR2) and Relaxin-3 (RLN3) can play an important role in tumor development through cytokine-cytokine receptor interactions and neuroactive ligand-receptor interactions [9]. RAD51-associated protein 1 (RAD51AP1) not only enhances tumor stemness but also exerts an influence on the tumor immune microenvironment. This dual impact represents a critical oncogenic mechanism associated with this gene. Subsequent investigations have substantiated the presence of such crosstalk involving this gene in various specific cancer types [10]. Likewise, Insulin-like growth factor 2 mRNA-binding proteins (IGF2BPs) have been linked to the tumor immune microenvironment and stemness across various human cancers. A noteworthy member of this family, IGF2BP3, plays a pivotal role in the maintenance and self-renewal of glioma stem cells [11]. All the aforementioned studies have underscored the existence of crosstalk between cellular stemness and the TIME, revealing the involvement of numerous genes in regulating this intricate process. In our current study, we leveraged extensive breast cancer histology data and experimental stemness data specific to breast cancer. Our aim was to elucidate the immune stemness genes associated with breast cancer, thereby offering novel targets for advancing breast cancer treatment strategies.

## Materials and methods

### Cell line construction and data source

The breast cancer cell line used in this study was the MDA-MB-231 cell line, a human breast cell line established from the pleural effusion of a 51-year-old white female with metastatic breast cancer, which had some cell stemness and was therefore selected as the experimental cell line for this study. Also in this study, a MDA-MB-231 cell line (named as LDLR) with stable high expression of low-density lipoprotein receptor (LDLR) and a MDA-MB-231 cell line with stable low expression of LDLR (named as shLDLR-1 and shLDLR-2) were constructed using cell transfection technology. The MDA-MB-231 cells were stably cultured at 37 °C in a CO_2_ incubator with 5% CO_2_ using DMEM medium containing 10% fetal bovine serum.

Autonomous sequencing data for this study were obtained from RNA-seq and Proteomics of four generation breast cancer cell lines (SP1–SP4). SP1–SP4 refer to four consecutive batches of breast cancer cells (i.e. 1st, 2nd, 3rd, 4th) produced during the process of cell cultivation, with the tumor stemness level gradually increasing. The RNA-seq of 3872 breast cancer cases and 113 normal breast tissues from four public databases (TCGA, GEO, ICGC and Metabric) were also collected in this study. For the reliability of the data, only patients with a follow-up time from 30 days to 5000 days were retained in this study, and 3132 breast cancer patients were finally enrolled. The RNA-seq data of 3132 cases used in this study were de-batched using the sva package and normalized in FPKM.

### Spheroid formation experiment

The spheroid formation experiment is mainly used in cancer stem cell research and human tumor cell research. Cells were cultured into ultra-low adhesion 96-well plates (Corning) at a density of 500 cells/well. Cells were cultured in DMEM/F12 (Gibco) supplemented with B27 (Invitrogen), 20 ng/ml epithelial growth factor (EGF, Sigma-Aldrich), 20 ng/ml basic fibroblast growth factor (bFGF, PeproTech) and 1% methylcellulose (Sigma-Aldrich). Cells were cultured for 10 days and each experiment was repeated three times. In serial sphere experiments, cells were cultured at a density of 5000 cells/well into ultra-low adhesion six-well plates (Corning) without methylcellulose during culture. After 1 week, individual cells from the digested spheres were used for the formation of the next generation of spheres. All results were photographed and counted under an inverted microscope (Olympus) on the spheres. In this study, we conducted transcriptome sequencing and proteomic sequencing on cells from the first generation (1st) to the fourth generation (4th) in the spheroid formation experiment, at a total of four time points, in order to identify genes that influence cell stemness.

### Western blotting experiment

Western blotting experiments in this study was mainly used to detect target proteins from protein mixtures and to quantitatively or qualitatively determine the expression of proteins in cells or tissues. Cells were lysed with RIPA buffer [150 mM NaCl, 0.5% sodium deoxycholate, 0.1% SDS, 1% NP40, and 50 mM Tris (pH 8.0)] and a protease inhibitor (Sigma-Aldrich) ice bath. Lysates were centrifuged at 12,000 rpm for 15 min. Proteins were then quantified by the Coomassie bright blue dye method. After boiling for 5 min with the sample buffer, an equal amount of cellular proteins was supersampled and separated in SDS-PAGE, and then transferred to a nitrocellulose membrane (Millipore). After blocking, the membrane was incubated with primary antibody at 4 °C overnight. The membrane was then incubated with HRP-conjugated secondary antibody for 1 h at room temperature. Color was developed using an enhanced chemiluminescence kit (Advansta) according to the manufacturer’s instructions. Each experiment was repeated three times. Therefore, we determined the expression of stemness-related markers (Oct4, CD44, EpCAM and Vimentin) in breast cancer cells with different stemness degrees by this experiment and could verify the protein expression of LDLR in our constructed cell lines.

### Clonogenic cell survival assay

In this study, the Clonogenic Cell Survival Assay was employed to assess cell proliferation capability. Cells were seeded at a density of 500 cells per well in 6-well plates. After 7–10 days of incubation, they were fixed using 4% paraformaldehyde, followed by staining with a 0.5% crystal violet solution (Sigma-Aldrich). Subsequently, the cells were rinsed and allowed to air-dry. Images of the stained plates were captured. The bound crystal violet was then dissolved with 50% glacial acetic acid solution and the absorbance was measured at 570 nm using a multimode plate reader (Perkin Elmer). Alternatively, the number of clonal colonies was calculated using ImageJ software. Each experiment was repeated three times. In this study, we used Clonogenic Cell Survival Assay to detect the proliferation ability of breast cancer under different modifications in order to reflect the stem proliferation ability of this breast cancer cells from the side.

### Flow cytometry sorting assay

In this study, FACSAria flow cytometry was used for aseptic sorting of breast cancer cell lines. Four clusters of breast cancer cells were sorted based on the LDLR expression on the surface of the tumor cells. The P2 cell cluster was LDLR-negative, P3 cell cluster was low LDLR, P4 cell cluster was medium LDLR, and P5 cell cluster was high LDLR. Finally, the spheroid formation experiment was applied to study the cell stemness ability of different LDLR expressing cell populations.

### Gene trend analysis and consistency clustering

In this study, we obtained gene and protein expression data from breast cancer cells at various time points spanning from SP1 to SP4, leveraging autonomously sequenced transcriptome and proteome data. Based on the built-in cutree_rows function in the pheatmap package [12], we performed clustering analysis on the transcriptome and proteome data of SP1, SP2, SP3, and SP4 and generated heatmaps. The specific analysis parameters used were as follows: scale = “row”, cluster_cols = FALSE, cutree_rows = 8. Using the aggregate function, we calculated the average expression values of SP1, SP2, SP3, and SP4 within each cluster separately, and then used the plot function to visualize the trend changes. Our objective was to identify clusters, from a macro perspective, that are most relevant to breast cancer stemness, meaning clusters where the average expression values of SP1, SP2, SP3, and SP4 show a gradual increase.

The molecules that exhibited an upward trend in both transcriptomic and proteomic trend analyses were retained for subsequent analysis. In total, we obtained 65 molecules (Additional file 1: Table S1) that met these criteria and were considered as breast cancer stemness-related genes. Based on the public RNA-seq data and survival data, 41 genes (Additional file 1: Table S2) were identified to be associated with breast cancer prognosis. Utilizing the ConsensusCluster package and the aforementioned set of 41 genes, we conducted stemness subtype classification on publicly available RNA-seq data from breast cancer patients. This categorization resulted in the classification of breast cancer into two distinct subtypes: one characterized by high stemness and the other by low stemness.

### Differential gene expression analysis and pathway enrichment analysis

In this study, differential gene expression analysis was performed for two subtypes of breast cancer based on limma package [13, 14]. We also performed GSEA and GSVA enrichment analysis based on the differential genes, and analyzed the pathway differences in immunity, metabolism, and cell death of different subtypes [15]. Specifically, the gene sets associated with immunity, metabolism, and cell death were obtained from Molecular Signatures Database (MSigDB database) and previous published articles (Additional file 1: Tables S3–S5) [16]. In this study, we compiled relevant pathway annotations sourced from the MSigDB database and previously published articles utilizing keywords related to research aspects such as metabolism, immunity, and more. These compiled gene sets were then constructed for further analysis [16]. Furthermore, we employed the ssGSEA algorithm to generate stemness scores based on the expression patterns of the 41 stemness genes. This approach allowed us to assess and quantify the disparities in stemness levels between the two identified subtypes.

### Gene pan-cancer analysis

In this study, we sought to provide a comprehensive insight into the pivotal roles and functional distinctions of genes in cancer. To achieve this, we conducted a pan-cancer functional analysis utilizing the TCGA pan-cancer data cohort. Our primary emphasis was placed on examining gene expression, methylation patterns, single nucleotide variations (SNV), and copy number variations (CNV). This holistic approach enabled us to gain a deeper understanding of how breast cancer stemness genes function across a spectrum of 33 known cancer types. The specific methods were similar with our previous studies [16, 17]. Notably, during the pan-cancer expression analysis and pan-cancer methylation analysis, we utilized TCGA’s adjacent non-tumor samples as control groups. We performed comparisons between each type of tumor sample and these controls to identify any abnormal increases or decreases in their transcription levels and gene methylation levels.

### Immune infiltration analysis

In this study, we assessed the immune microenvironment profile of breast cancer by leveraging RNA-seq data and employing the Estimate algorithm. To further evaluate immune cell infiltration in breast cancer, we utilized the TIMER2 online analysis tool [18]. Additionally, we investigated disparities in the expression of immune checkpoint molecules within various breast cancer subtypes. Ultimately, we delineated the immune characteristics associated with stemness genes using the aforementioned methodologies.

### Analysis of LDLR protein levels

In this study, we utilized the CPTAC proteomics database to conduct a differential protein expression analysis of LDLR [19]. Furthermore, we obtained immunohistochemistry (IHC) experimental data for LDLR in breast cancer from the HPA database [20]. This allowed us to perform a comprehensive protein-level analysis of LDLR expression in breast cancer.

### Drug sensitivity analysis and clinical characterization

In this study, we predicted the sensitivity of commonly used breast cancer chemotherapeutic drugs across various breast cancer subtypes using the oncopredict package [21] in conjunction with the GDSC2 pharmacogenetic dataset. We performed KM survival analysis to assess the impact of univariate gene expression on the prognosis of breast cancer patients. Subsequently, we conducted lasso regression analysis to identify nine immune stemness genes that exhibited higher significance. To ensure consistency with prior studies [22, 23], we divided the breast cancer data from the Metabric database into two subsets comprising 660 and 659 cases using the caret package. A total of 660 samples from the Metabric database were utilized as the training dataset for the prognostic model. Subsequently, the remaining 659 samples from the Metabric database were designated as internal validation dataset (1) All samples from the Metabric database were combined to form internal validation dataset (2) All samples from the TCGA database were used as the external validation dataset 1. Lastly, samples from GEO and ICGC database were combined and considered as the external validation dataset2. In the training dataset, predict algorithm in R helps establish a novel 9-gene prognostic model. Similar method was utilized in internal validation datasets and external validation datasets to assess the risk scores of each sample. Importantly, samples derived from training dataset, internal validation datasets, and external validation datasets were all divided into two subgroups (i.e. high- and low-risk subgroup) based on the median risk score of training dataset. We constructed high and low risk scores based on gene expression and analyzed the prognosis of the two groups of patients with high and low risk scores with respect to the expression of immune stemness genes. This further demonstrates the predictive role of nine immune stemness genes on the prognosis of breast cancer patients.

### Single-cell RNA-seq analysis

In our study, we utilized single-cell RNA-seq data from breast cancer, sourced from GSE161529 in the GEO database, to investigate the role of LDLR within the immune microenvironment. This dataset encompassed a total of 421,761 cells originating from 52 patients. To narrow our focus to the breast cancer component, we selected a subset comprising 125,800 cells for subsequent analysis.

We conducted quality control procedures and cell clustering on the single-cell RNA-seq data, mirroring methods employed in previous studies [24]. Cellular annotation was performed using SingleR, and cell cycle prediction was executed using Tricycle [25]. Finally, we examined the expression patterns of stemness genes in conjunction with LDLR across different cell populations.

### Statistical analysis

The data analyzed in this study were analyzed using R studio software. The correlation test was performed using the Spearman correlation test. Non-parametric test (Kruskal–Wallis test) was used for the analysis of differences between samples. p < 0.05 was considered as statistically significant statistical results.

## Results

### Cell stemness-related genes in breast cancer

**Fig. 1:**
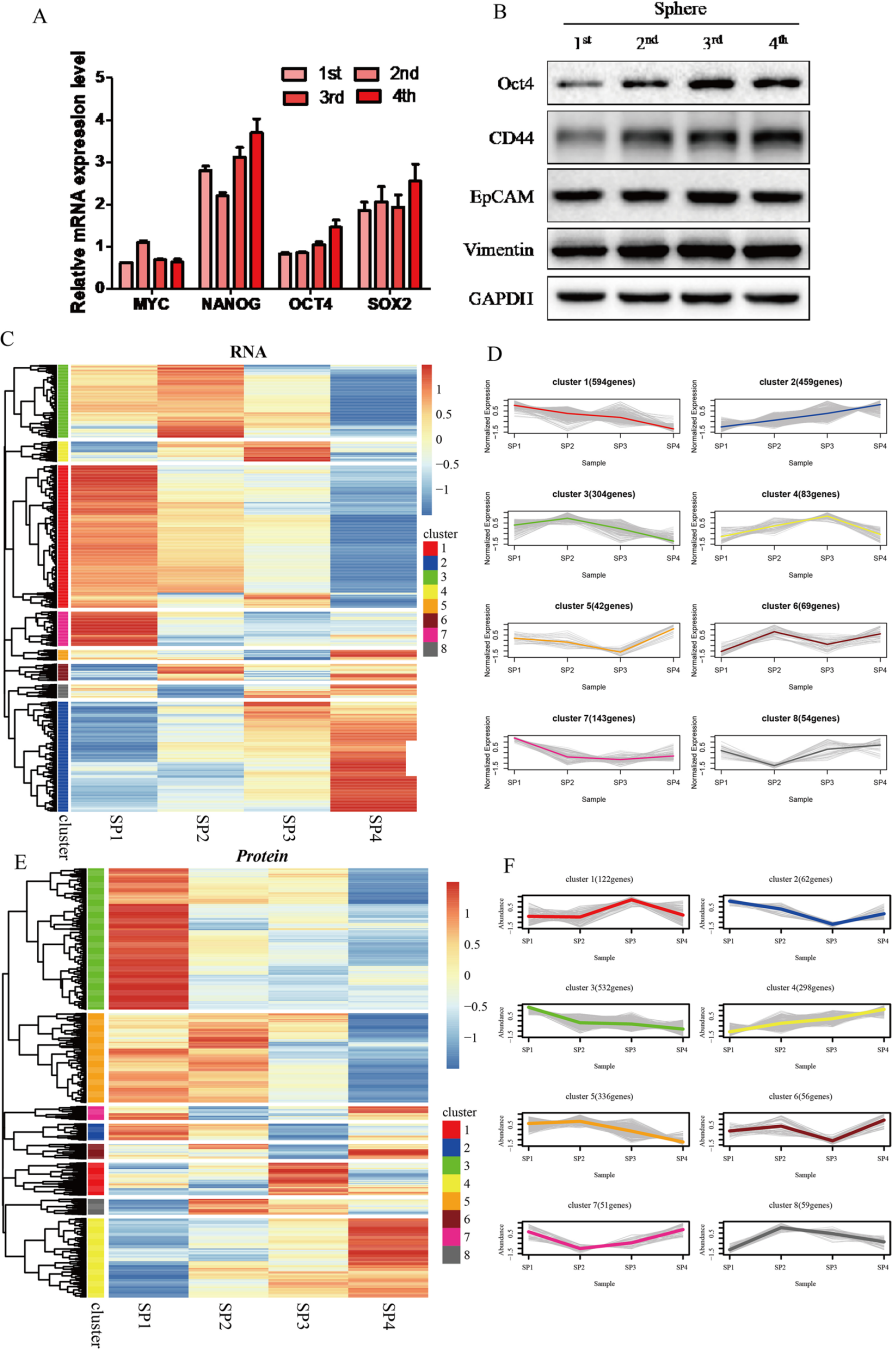
Cell stemness-related genes CD44 and Oct4 promote the formation of breast cancer spheroid. **A** mRNA expression levels of stemness-related markers (MYC, NANOG, OCT4 and SOX2) in SP1–SP4. **B** Protein contents of stemness-related markers (CD44, OCT4, EpCAM and Vimentin) in SP1–SP4. **C** The gene heatmap based on RNA-seq of four cell clusters. **D** Gene trend analysis based on RNA-seq of four cell clusters. **E** The gene heatmap based on proteomics of four cell clusters. **F** Gene trend analysis based on proteomics of four cell clusters

In this study, we collected breast cancer cells at 1st, 2nd, 3rd, and 4th which were numbered SP1, SP2, SP3 and SP4 in the spheroid formation experiment. Our findings revealed a gradual increase in the mRNA expression levels of stemness-related markers, OCT4 and SOX2, in breast cancer cells over time (Fig. 1A). Concurrently, there was a gradual rise in the protein content of stemness-related markers, CD44 and OCT4, at the protein expression level (Fig. 1B). RNA-seq was performed on four clusters of cell lines, and the genes were clustered (Fig. 1C) and analyzed for gene trends (Fig. 1D). The results unveiled that 459 genes within cluster 2 exhibited a progressive increase in expression levels over time. Proteomics was performed on four clusters of cell lines, and the proteins were clustered (Fig. 1E) and analyzed for protein trends (Fig. 1F). The results demonstrated that within cluster 4, 298 proteins exhibited a gradual increase in their protein expression levels as time progressed. In this study, we observed a certain association between the elevated expression of the mentioned genes and the growth of breast cancer spheroids. It can be assumed that these genes might play a role in promoting breast cancer stemness and could be considered as potential contributors to stemness in breast cancer.

### Genetic characteristics of breast cancer stemness genes

**Fig. 2:**
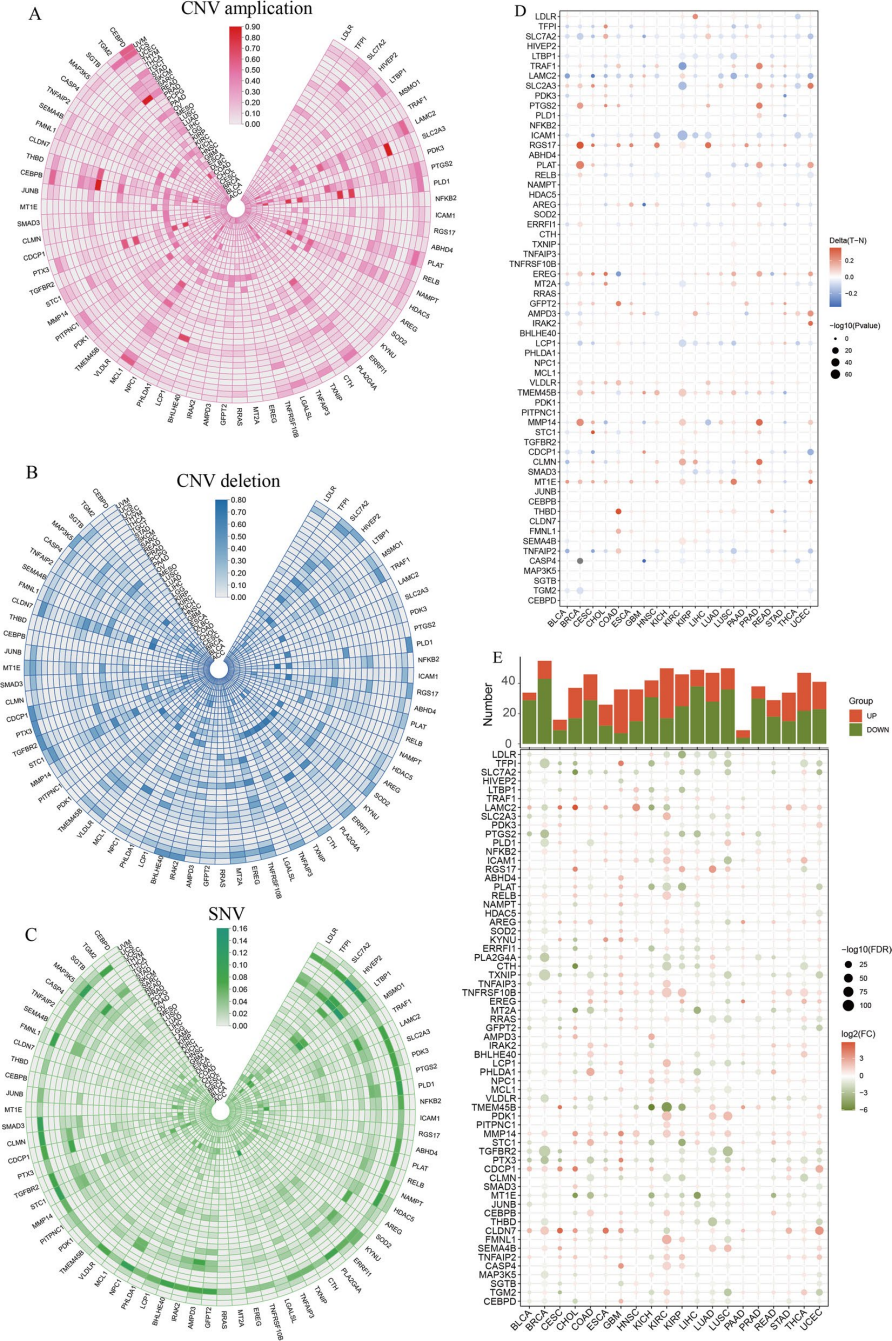
Gene pan-cancer analysis of breast cancer stemness genes. **A** CNV amplification of 65 stemness genes in pan-cancer. **B** CNV deletion of 65 stemness genes in pan-cancer. **C** SNV of 65 stemness genes in pan-cancer. **D** Methylation between tumor and normal of 65 stemness genes in pan-cancer. **E** Gene expression of 65 stemness genes in pan-cancer. F Pathway enrichment analysis of 65 stemness genes in pan-cancer

**Fig. 3:**
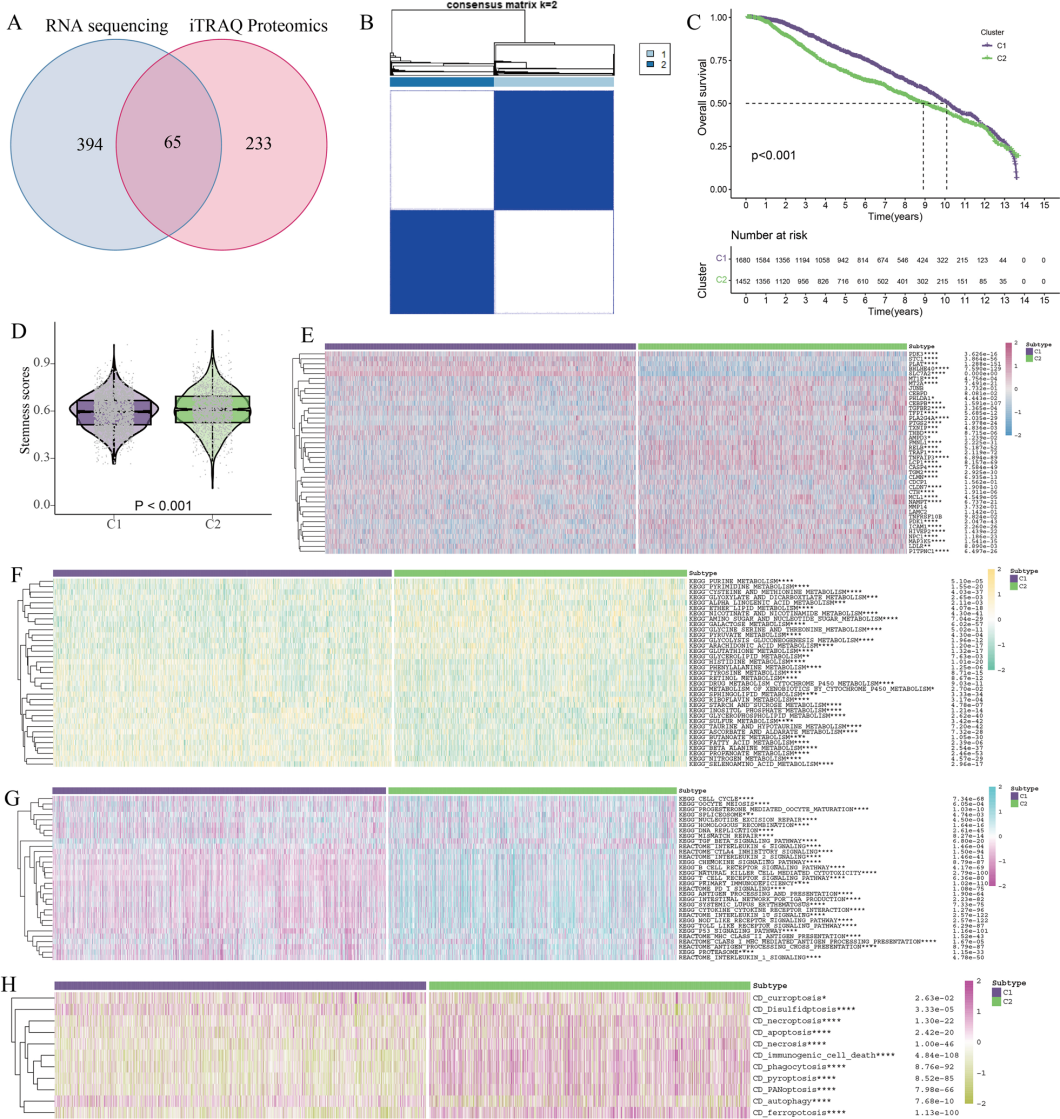
Stemness subtypes and key stemness gene in breast cancer. **A** 65 genes were obtained by intersection of RNA-seq and Proteomics. **B** Identification of C1 and C2 subtypes of breast cancer based on 41 stemness genes. C KM survival analysis of C1 and C2 subtypes. **D** The stemness scores of the C1 and C2 subtypes based on ssGSEA algorithm. **E** The expression of 41 stemness genes of the C1 and C2 subtypes. *p < 0.05; **p < 0.01; ***p < 0.001; ****p < 0.0001. **F** Metabolic pathway enrichment analysis of the C1 and C2 subtypes. *p < 0.05; **p < 0.01; ***p < 0.001; ****p < 0.0001. **G** Immune pathway enrichment analysis of the C1 and C2 subtypes. *p < 0.05; **p < 0.01; ***p < 0.001; ****p < 0.0001. **H** Cell death pathway enrichment analysis of the C1 and C2 subtypes. *p < 0.05; **p < 0.01; ***p < 0.001; ****p < 0.0001

The RNA sequencing data indicated that cluster2, comprising 459 genes, exhibited an increasing expression trend with the SP generation. Similarly, the proteomic sequencing data revealed a similar trend in cluster4, which contains 298 genes encoding proteins. Subsequently, an intersection analysis was conducted between the subgroups of cluster2 from RNA sequencing and cluster4 from proteomic sequencing, resulting in a total of 65 shared genes being considered as breast cancer stemness-related genes (Fig. 3A, Additional file 2: Fig. S1). We performed pan-cancer analysis of 65 genes. Most genes had lower CNV amplification (Fig. 2A) and deletion (Fig. 2B) and lower SNV (Fig. 2C) in multiple tumors, especially in breast cancer. Additionally, we observed increased methylation (Fig. 2D) and reduced expression (Fig. 2E) of the majority of the 65 genes in cancer tissue in comparison to adjacent non-tumor tissue. The aforementioned findings indicated that the 65 stemness genes identified in breast cancer exhibited a more consistent gene expression pattern within cancer tissue, suggesting their potential relevance to the immune microenvironment.

### Stemness subtypes and key stemness gene in breast cancer

We performed KM survival analysis of 65 genes based on 3132 breast cancer data from public databases, and we found that 41 genes were associated with the prognosis of breast cancer (Additional file 3: Fig. S2). Based on the 41 stemness genes, we applied consensus clustering to classify breast cancer into C1 and C2 subtypes (Fig. 3B). KM survival analysis showed that the prognosis of C1 subtype was better than that of C2 subtype (Fig. 3C). We incorporated 41 stemness genes expressions into the ssGSEA algorithm to calculate the stemness scores for each sample, and the stemness scores was higher in the C2 subtype than in the C1 subtype (Fig. 3D). We examined the expression of the 41 stemness genes in both subtypes, revealing that 36 of these genes exhibited higher expression in subtype C2 compared to subtype C1 (Fig. 3E). Subsequently, we conducted a pathway enrichment analysis using breast cancer expression data from both subtypes. The outcomes indicated that the C2 subtype displayed more attenuated metabolic responses than the C1 subtype, particularly in relation to lipid metabolism (Fig. 3F). Moreover, the C2 subtype exhibited more pronounced cell cycle pathways than the C1 subtype (Fig. 3G), suggesting a higher proliferative capacity in the former. Furthermore, the C2 subtype showed a greater inclination towards specific cell death patterns, particularly those associated with the immune system. Conversely, the C1 subtype demonstrated a more robust autophagic pathway compared to the C2 subtype, hinting at a reduced susceptibility to normal cell death in response to conventional treatments (Fig. 3H). Collectively, these results strongly indicate that the C2 subtype displays an elevated level of malignancy compared to the C1 subtype, highlighting how alterations in stemness gene expression can contribute to the heightened malignancy of breast cancer.

### Effect of breast cancer stemness genes on immune microenvironment

**Fig. 4:**
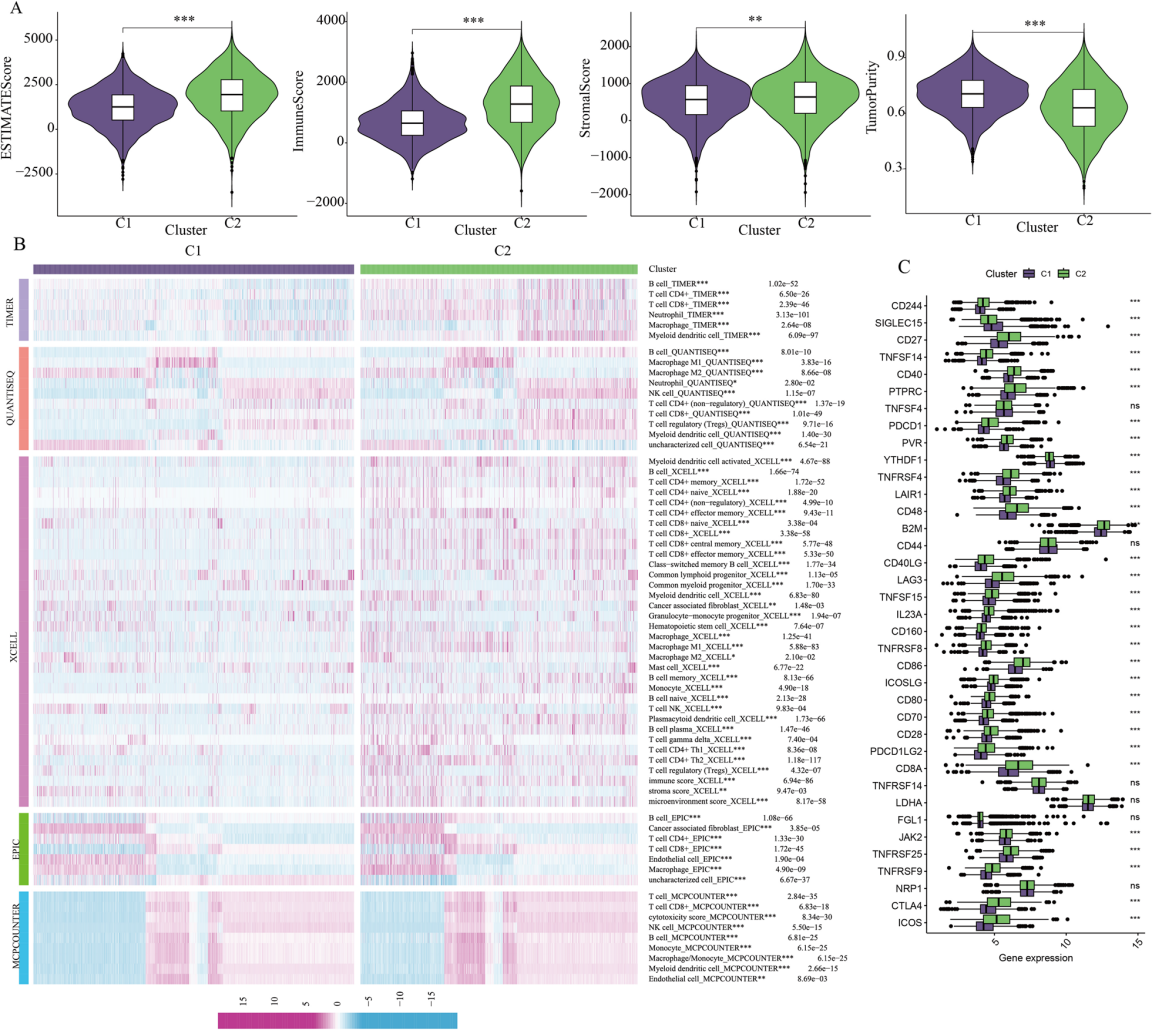
Effect of breast cancer stemness genes on immune microenvironment. **A** Immune microenvironment assessment of the C1 and C2 subtypes based on Estimate algorithm. *p < 0.05; **p < 0.01; ***p < 0.001; ****p < 0.0001. **B** Immune cell infiltration analysis for the C1 and C2 subtypes based on five algorithms: Timer, Quantiseq, xCell, EPIC and MCPCOUNTER. *p < 0.05; **p < 0.01; ***p < 0.001; ****p < 0.0001. **C** Expression of immune checkpoints in the C1 and C2 subtypes. *p < 0.05; **p < 0.01; ***p < 0.001; ****p < 0.0001

The categorization of breast cancer into subtypes C1 and C2, based on the expression of 41 stemness-associated genes, provides valuable insights into the roles of these genes in breast cancer development and microenvironmental changes. In this study, we conducted an analysis of the immune microenvironment related to these two breast cancer subtypes. Utilizing the Estimate algorithm, we observed that the C2 subtype displayed a higher Estimate score, indicating increased immune cell infiltration and lower tumor purity (Fig. 4A). This suggests that the C2 subtype exhibits greater immune infiltration compared to the C1 subtype. Subsequently, we conducted immune cell infiltration analysis for both breast cancer subtypes using five different algorithms: Timer, Quantiseq, xCell, EPIC, and MCPCOUNTER. The results consistently revealed higher levels of immune cell infiltration in the C2 subtype (Fig. 4B). Additionally, we compared the expression of immune checkpoint molecules in the two subtypes and observed higher expression levels of CTLA4 and LAG3 in the C2 subtype (Fig. 4C). This implies a more pronounced suppression of the immune microenvironment in the C2 subtype. Collectively, these findings indicate that the C2 subtype exhibits greater immune infiltration and a more pronounced immune cell suppression when compared to the C1 subtype.

**Fig. 5:**
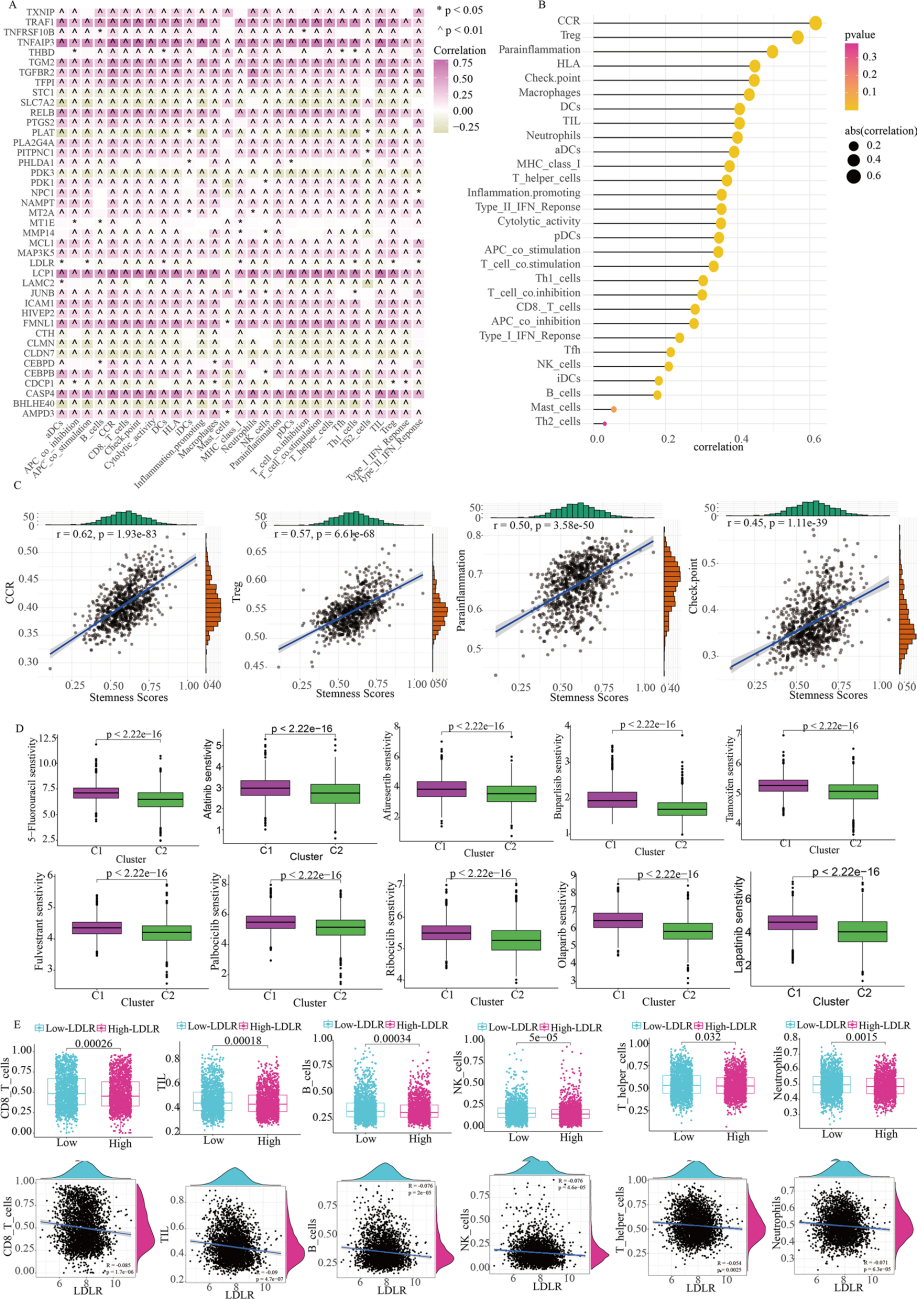
Immune stemness genes and immune function of LDLR. **A** Relationship between immune cell pathways and 41 immune stemness genes. *p < 0.05; ^p < 0.05. **B** Relationship between immune cell infiltration and Stemness scores. **C** Relationship between Stemness scores and CCR, Treg, parainflammation and checkpoint. **D** Drug sensitivity analysis between the C1 and C2 subtypes. **E** Relationship between expression of LDLR and the cellular infiltration of CD8_T cells, TIL cells, B cells, NK cells, T helper cells and neutrophils

Moreover, we assessed the relationship between the 41 stemness genes and immune cell infiltration in breast cancer using the ssGSEA algorithm. It was evident that the majority of stemness genes played a role in promoting the development of immune cell infiltration (Fig. 5A). The stemness scores with 41 breast cancer stemness genes was generally associated with chemokine receptor (CCR), Treg cell infiltration, Parainflammation, and immune checkpoints (Fig. 5B, C). Furthermore, our drug sensitivity analysis indicated that the C2 subtypes exhibited greater sensitivity than the C1 subtypes to commonly used breast cancer drugs. This suggests that the C2 subtype may offer more therapeutic options for breast cancer treatment (Fig. 5D). In summary, 41 stem genes have shown strong immune functions and could be considered as immune stemness genes.

### Establishment of an immune stemness-based prognostic model

**Fig. 6:**
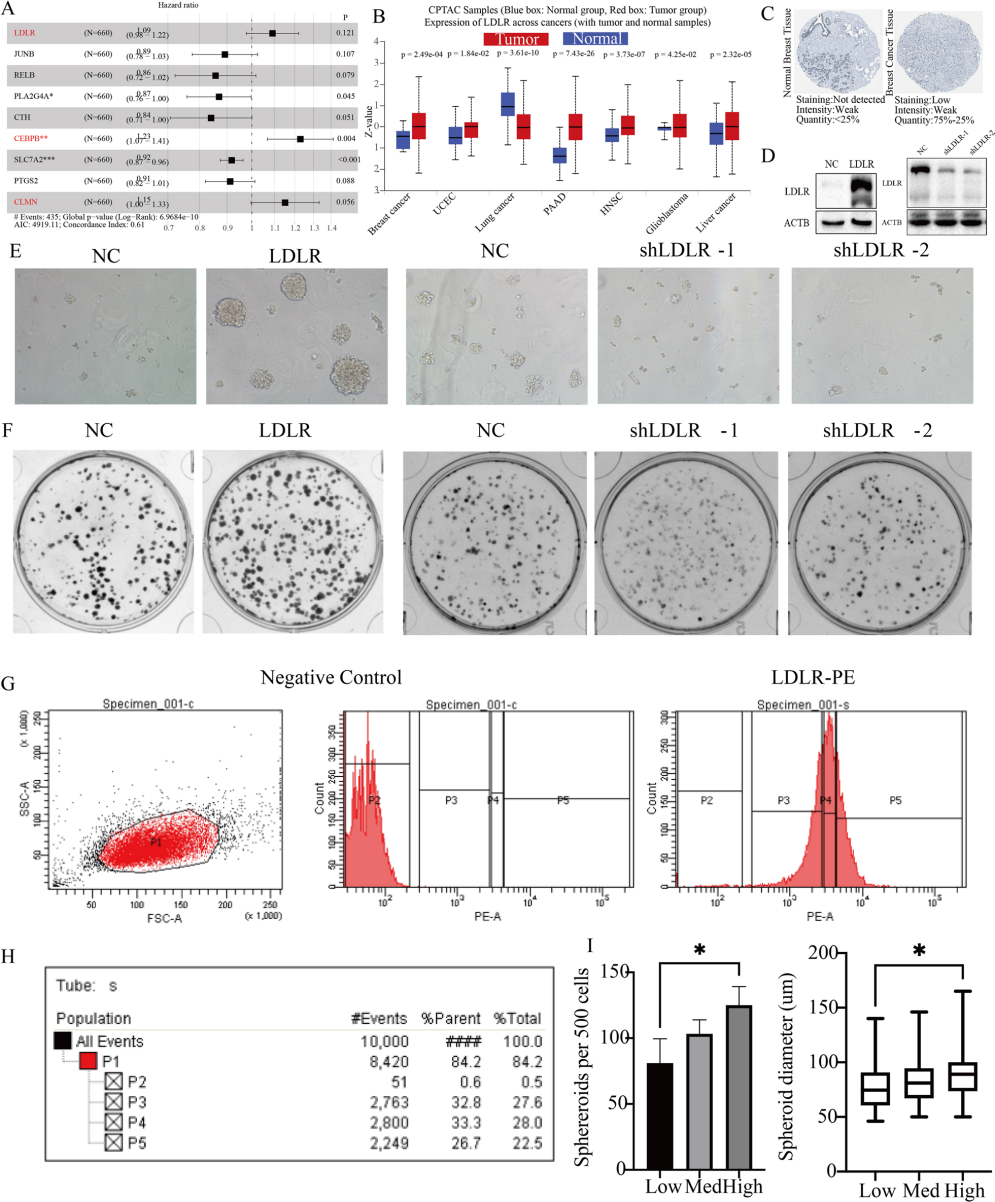
Regulation of breast cancer cell stemness by LDLR. **A** Hazard ratio for 9 immune stemness genes in survival models. **B** Expression of LDLR in a variety of tumor tissues in CPTAC. **C** Protein expression of LDLR in breast cancer in HPA. **D** Construction of MDA-MB-231 cell lines with high and low expression of LDLR. **E** Sphere formation experiment in MDA-MB-231 cell lines with high and low expression of LDLR. **F** Clonogenic Cell Survival Assay MDA-MB-231 cell lines with high and low expression of LDLR. G Four clusters of cells were obtained by flow cytometry sorting: P2–P5. **H** Proportion of 4 clusters of cells obtained by flow cytometry. **I** The sphereroids diameter and sphereroids per 500 cells in P3–P5. *p < 0.05; **p < 0.01; ***p < 0.001; ****p < 0.0001

In this study, we screened 14 immune stemness genes by lasso regression analysis using 41 immune stemness genes (Additional file 4: Fig. S3A, B). We employed a training set comprising 660 breast cancer cases from the Metabric database to build a survival model utilizing the multivariate Cox survival regression algorithm for the 9 immune stemness genes. Subsequently, we stratified the patients into two groups, categorizing them as either high or low risk, based on their median score (Additional file 5: Fig. S4A, B). The expression distributions of 9 model genes between different risk groups were shown in Additional file 5: Fig. S4C. The high-risk group exhibited a notably lower survival time compared to the low-risk group (Additional file 5: Fig. S4D). Based on the median risk score of training dataset, the samples in internal validation dataset 1 and 2 were similarly divided into high- and low-risk groups (Additional file 6: Fig. S5A, B, Additional file 7: Fig. S6A, B). Clearly, there are a higher number of deceased samples in the high-risk group of the internal validation datasets. Similarly, the expression traits of 9 model genes in internal validation dataset 1 and 2 were also shown in Additional file 6: Fig. S5C and Additional file 7: Fig. S6C. More importantly, in both internal validation sets 1 and 2, the low-risk group demonstrated a clear survival advantage (Additional file 6: Fig. S5D, Additional file 7: Fig. S6D). A truly exceptional prognostic model not only necessitates validation within internal datasets but also calls for rigorous testing across numerous external datasets. Only through this extensive validation can our immune stemness model achieve a wider range of applicability and practicality. Subsequently, we conducted external validation of the model using previously established external validation sets 1 and 2. In alignment with the training dataset, we categorized the patients into two risk groups, based on the median risk score of training dataset (Additional file 8: Fig. S7A, B, Additional file 9: Fig. S8A, B). In the external validation set, the model genes also exhibited distinct expression distribution characteristics between high- and low-risk groups (Additional file 8: Fig. S7C, Additional file 9: Fig. S8C). Likewise, compared to the high-risk group, the low-risk group demonstrates a significant survival advantage (Additional file 8: Fig. S7D, Additional file 9: Fig. S8D). Finally, we identified three immune stemness genes (LDLR, CEBPB, CLMN) as the major risk genes for breast cancer (Fig. 6A). Among them, LDLR is an important membrane protein, which is associated with lipoprotein metabolism. In breast cancer, high expression of LDLR inhibits the cellular infiltration of CD8_T cells, TIL cells, B cells, NK cells, T helper cells and neutrophils (Fig. 5E). This study also identified LDLR as a predominant risk gene in various cancers through pan-cancer analysis. Additionally, it was observed that LDLR was associated with reduced survival time in breast cancer patients across multiple breast cancer cohorts (Additional file 10: Fig. S9). In addition, LDLR expression was increased in older breast cancers with poorer pathological subtypes and promoted lymph node metastasis and distant metastasis in breast cancers (Additional file 11: Fig. S10). In summary, we identified LDLR as a more malignant immune stemness gene in breast cancer, which can promote malignant progression of breast cancer. And due to the membrane protein properties of LDLR, LDLR also has some diagnostic and therapeutic value.

### Regulation of breast cancer cell stemness by LDLR

Our study identified the role of LDLR in the development of breast cancer through lasso and complex clinical prognostic analysis. At the proteomic level, LDLR has higher protein expression in a variety of tumor tissues (Fig. 6B). Furthermore, the results of IHC provided additional validation for the elevated protein expression of LDLR in breast cancer (Fig. 6C). To further explore the stemness function of LDLR, we constructed MDA-MB-231 cell lines with high LDLR expression (LDLR) and MDA-MB-231 cell lines with low LDLR expression (shLDLR-1 and shLDLR-2) (Fig. 6D). Sphere formation experiment showed that LDLR cell lines had stronger sphere-forming ability than normal cells, while shLDLR-1 and shLDLR-2 cell lines had weaker sphere-forming ability than normal cells (Fig. 6E, Additional file 12: Fig. S11A, B). Clonogenic assay showed that the LDLR cell line had a stronger proliferation ability than normal cells, while the shLDLR-1 and shLDLR-2 cell lines had a weaker proliferation ability than normal cells (Fig. 6F, Additional file 12: Fig. S11C, D). In summary, gene expression of LDLR regulates the stemness ability of breast cancer cells. Since LDLR is a more common membrane protein receptor in various tissues, cell sorting of breast cancer cell lines were performed by flow cytometry. In this study, we identified the target cell populations (P1) based on their characteristics in SSC-A and FSC-A scatter plots. We distinguished four distinct cell clusters, which include a cluster with no LDLR expression (P2), a cluster with low LDLR expression (P3), a cluster with medium LDLR expression (P4), and a cluster with high LDLR expression (P5) (Fig. 6G, H). Sphere formation experiments were conducted on the three clusters of breast cancer cells (P3–P5), revealing that spheroid diameter and the number of spheroids per 500 cells were greater in P5 compared to P4 and P3. The sphere-forming capability of breast cancer cells was enhanced with increasing LDLR expression (Fig. 6I). This further confirms that LDLR promotes enhanced stemness of breast cancer cells and serves as an important membrane protein to further classify breast cancer cells.

### Gene function of LDLR in pan-cancer

**Fig. 7:**
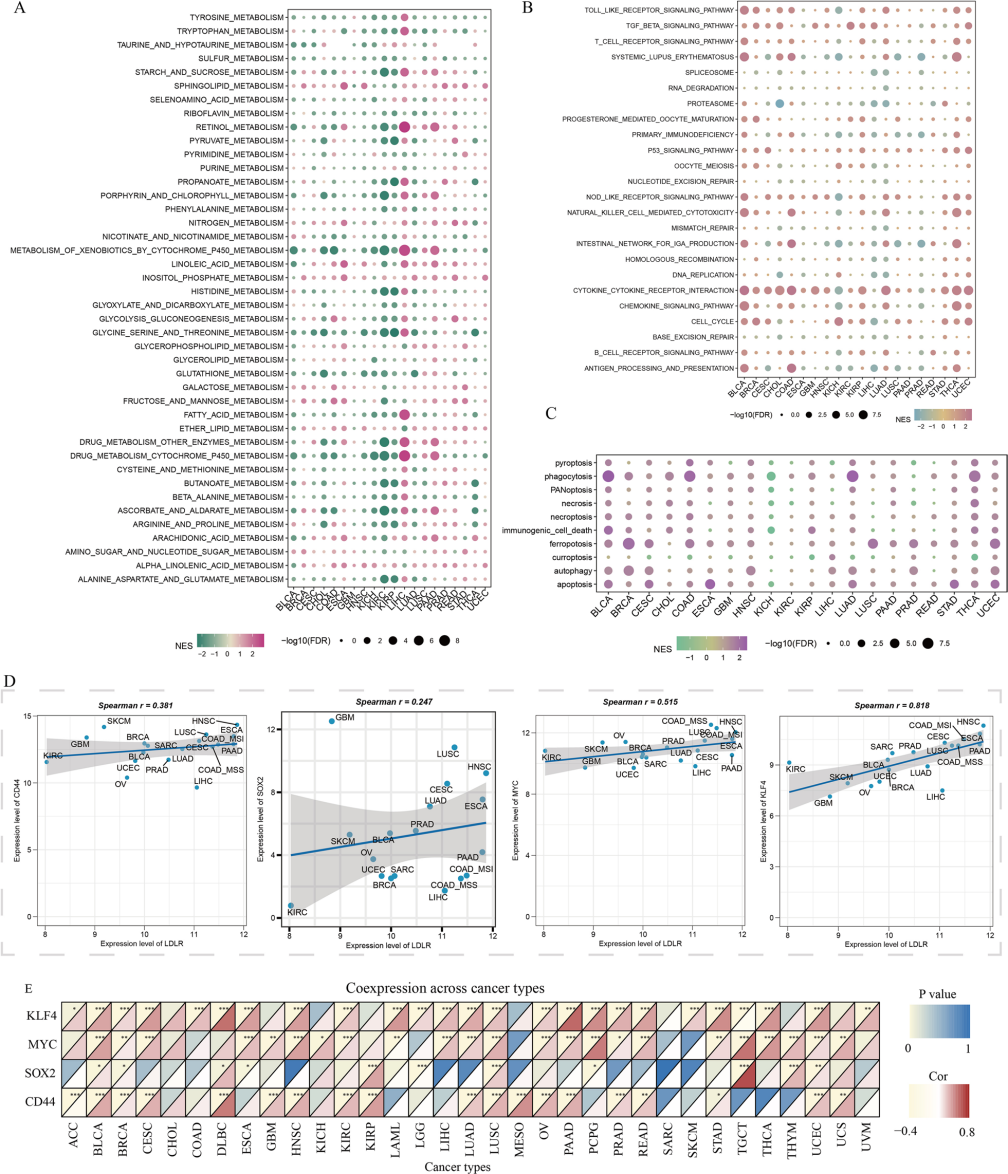
Gene function of LDLR in pan-cancer. **A** Metabolic pathways enrichment analysis of LDLR in pan-cancer. **B** Immune microenvironmental pathways enrichment analysis of LDLR in pan-cancer. **C** Cell Death pathways enrichment analysis of LDLR in pan-cancer. **D** The correlation of common stemness markers (SOX2, CD44, MYC and KLF4) and LDLR in pan-cancer. **E** The correlation of common stemness markers (SOX2, CD44, MYC and KLF4) and LDLR in different cancers. *p < 0.05; **p < 0.01; ***p < 0.001; ****p < 0.0001

In this study, the function of LDLR was systematically analyzed and studied, based on the pan-cancer analysis. In terms of metabolic pathways, LDLR can inhibit drug metabolic processes in a variety of tumors (Fig. 7A). In terms of immune microenvironmental pathways, LDLR promoted immune inflammatory processes in most tumors, and in breast cancer LDLR promoted cell cycle processes and inhibits tumor immunogenesis (Fig. 7B). In terms of cell death, LDLR promoted many cell death processes in a variety of tumors (Fig. 7C). In breast cancer, LDLR promoted the ferropotosis and autophagy (Fig. 7C). In this study, we analysed the correlation between LDLR expression and the expression of common stemness genes (SOX2, CD44, MYC and KLF4) in the pan-cancer. The results showed a positive and statistically significant correlation between LDLR expression and the expression of common stemness genes in breast cancer. Moreover, LDLR expression was positively correlated with the expression of common stemness genes in most types of tumours (Fig. 7D, E). And LDLR was associated with more immune cell infiltration, especially in breast cancer where LDLR inhibited more immune cell infiltration (Additional file 13: Fig. S12). In conclusion, we suggest that LDLR is a more obvious immune gene in tumors and has the ability to regulate the cell stemness and immune microenvironment in breast cancer.

### The immune stemness function of LDLR in scRNA-seq of breast cancer

**Fig. 8:**
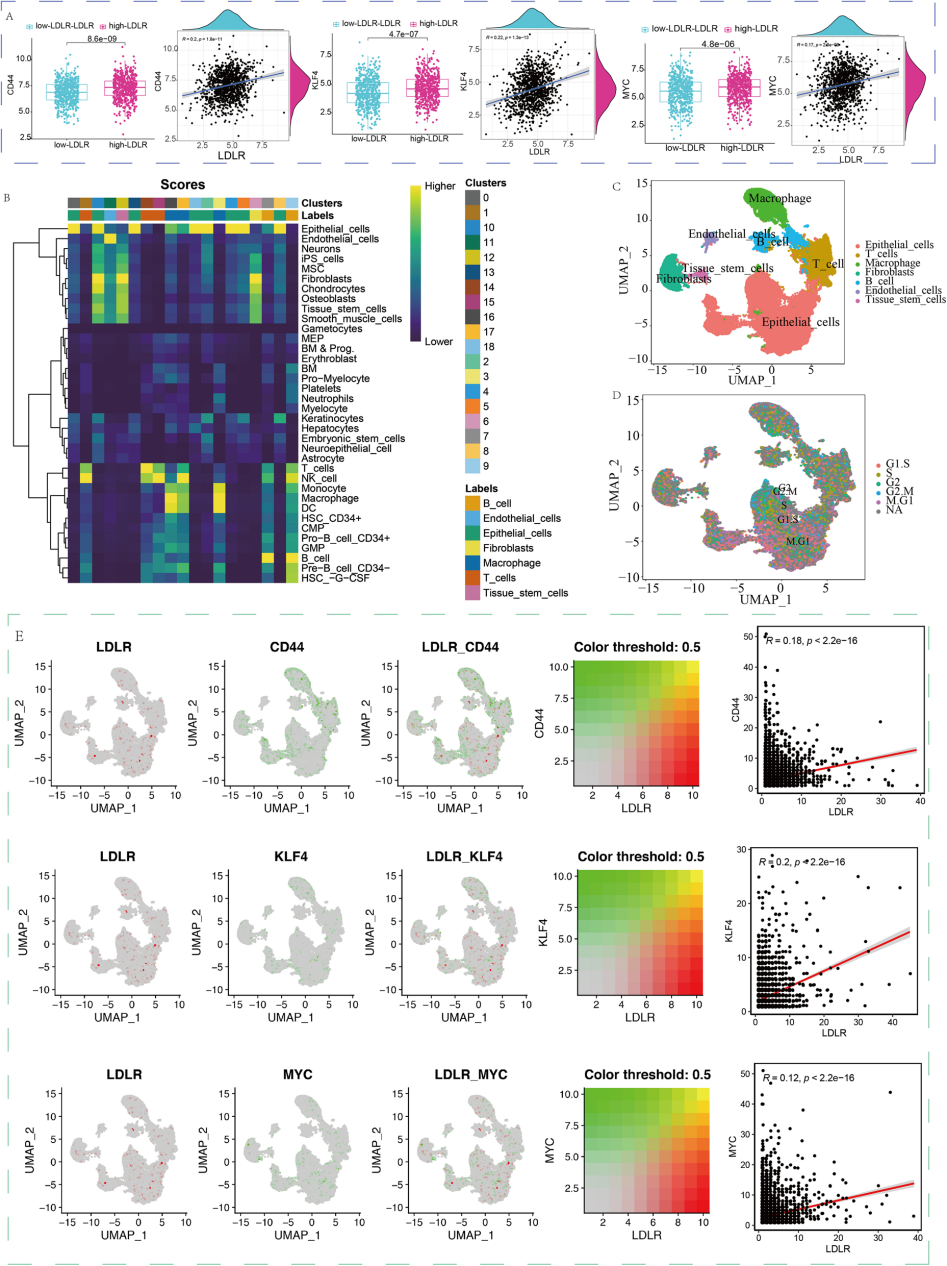
The immune stemness function of LDLR in scRNA-seq. **A** The correlation between common stemness markers (CD44, MYC and KLF4) and LDLR in scRNA-seq. **B** Seven class cell subgroups in scRNA-seq of breast cancer. **C** Distribution of 7 class cell subgroups in the scRNA-seq based on UMAP. **D** Distribution of cell cycle cells in the scRNA-seq based on UMAP. **E** The distribution of LDLR and stemness marker (CD44, KLF4, and MYC) expression in scRNA-seq based on UMAP

In this study, the breast cancer data from the scRNA-seq were quality controlled and standardized (Additional file 14: Fig. S13A–C). The standardized single-cell transcriptome has good homogeneity and can be used for subsequent data analysis (Additional file 15: Fig. S14A–C). We applied the SingleR algorithm for cell clustering and cell annotation and obtained 18 cluster cell subgroups (Additional file 15: Fig. S14D, Additional file 16: Fig. S15). We applied the Tricycle algorithm for cell cycle classification of the 18 cell subgroups (Additional file 15: Fig. S14E). The results of this study showed that tissue cells with high LDLR expression in breast cancer tissues had higher expression of CD44, KLF4, and MYC, demonstrating that tissue cells with higher LDLR expression had stronger stemness (Fig. 8A). We then annotated the 18 cell subgroups and grouped the 18 cell subgroups into 7 class cell subgroups (Fig. 8B). These 7 class cell subgroups were T cells, B cells, epithelial cells, fibroblasts, endocrine cells, macrophages, and tissue stem cells (Fig. 8C). In this study, the majority of epithelial cells were considered to be tumor cells and cell cycle analysis showed more cells in the epithelial tissue in the dividing phase of the cell cycle (M phase) (Fig. 8D). Our results also revealed that there were more cells with high LDLR expression in epithelial cells and less in T and B cells. Moreover, some of the epithelial cells with high LDLR expression were highly expressed in stemness marker genes (CD44, KLF4, and MYC), and there is a certain correlation (Fig. 8E). The above results indicated that most of the tumor cells in tumor tissues had highly expression of LDLR and strong cell stemness. In contrast, the majority of immune cells in tumor tissues did not express more LDLR, which might be an important reason for the immunosuppression of breast cancer tumor microenvironment, suggesting from the side that LDLR as a breast cancer immune stemness gene can promote the deterioration of immune microenvironment in breast cancer.

## Discussion

Breast cancer is currently the most prevalent malignancy with a low survival rate and a high recurrence rate. Breast cancer cells have different metabolic patterns and energy requirements compared to normal breast cells. Usually breast cancer cells are surrounded by a large number of adipocytes and can obtain more adipose from adipocytes for cancer cell proliferation and metastasis [26]. Therefore, obesity and hyperlipidemia are important risk factors for the development of breast cancer and predispose breast cancer cells to develop some drug resistance [27, 28]. Breast cancer patients often show increased levels of cholesterol, low-density lipoprotein (LDL), and triglycerides in the blood, which makes targeting lipid metabolism an important potential targeting pathway [29, 30]. Key metabolic enzymes involved in fatty acid synthesis and oxidation can inhibit breast cancer cell proliferation, invasion, and metastasis by affecting tumor cell viability [31]; control breast cancer cell proliferation by affecting apoptosis, arresting the cell cycle, and preventing migration [32]; and activate oncogenic signals by affecting some metabolite formation. And lipid metabolism in tumor cells interacts extremely closely with the tumor microenvironment, and exogenous fatty acids promote cancer progression and survival [33]. In addition LDL, a common lipoprotein in the blood, can also affect the sensitivity of breast cancer to radiotherapy [34]. And LDL can affect a variety of malignant biological behaviors of breast cancer cells, in addition different phenotypes of breast cancer cells have different lipoprotein input and storage differences [35]. Ox-LDL is also present at high levels in the blood of tumor patients and can induce DNA structural changes and decreased DNA repair in breast cancer cells, resulting in altered breast cancer phenotypes [36]. This all suggests that elevated blood LDL and enhanced adipose metabolism in breast cancer contribute to increased malignancy of breast cancer.

LDLR, a common LDL receptor, binds to LDL and transports it into cells via endocytosis, which facilitates the adipose metabolism of cancer cells. More studies have found that LDLR has high expression in breast cancer, which promotes elevated blood cholesterol in breast cancer patients and leads to poor prognosis in breast cancer patients [37, 38]. High expression of LDLR results in higher uptake of LDL in the blood by breast cancer cells, which facilitates increased metabolism and increases the malignancy of breast cancer [39]. Ingested LDL promotes an increase in cholesterol in cancer cells and an increase in 27-hydroxycholesterol, which facilitates the proliferation of breast cancer cells and epithelial mesenchymal transition (EMT) [40, 41]. All of these indicate that an increase in LDLR is an important marker of poor prognosis in breast cancer. In the present study, LDLR was found to have a crosstalk role in promoting breast cancer cell stemness as well as inhibiting the immune microenvironment of breast cancer. As an important lipid metabolism receptor, LDLR has a certain amount of expression on the surface of immune cells in breast cancer and microenvironment. Our findings revealed that LDLR has high expression in breast cancer and can promote the stemness ability of breast cancer. Moreover, breast cancer cells with high LDLR expression can affect the tumor immune microenvironment and lead to immunosuppression in the tumor microenvironment, which are high expression of immune checkpoints and reduced infiltration of immune killer cells. In summary, we suggest that LDLR is an important immune stemness gene with the dual function of inducing stemness to suppress immunity. High expression of LDLR in breast cancer induces increased malignancy of breast cancer, which in turn leads to metastasis and recurrence. It is also noteworthy that the breast cancer microenvironment also has a small number of immune cells with high LDLR expression, suggesting that high LDLR expression in immune cells can promote the function of immune cells. However, tumor cells have higher LDLR expression, which makes immune cells lack less energy source in the tumor microenvironment. Therefore, reversing LDLR distribution in the tumor microenvironment may be an important approach to treat breast cancer.

Among LDLR, the common giant receptors are LRP1, LRP1B and LRP2, three proteins with strong structural homology but with large differences in cellular dynamics and expression [42]. The low expression of LRP2 in some breast cancers leads to a decrease in the activation of its nuclear receptor VDR, which promotes the proliferative process of breast cancer [43]. And LRP2 mRNA has been detected at considerably high levels in invasive tumors. Considerably high levels were detected in invasive breast cancer with high variability [44]. LRP1 is an important LDLR-related protein expressed in a large number of immune cells. LRP1 reduces the abundance of TNF receptors on the cell surface to suppress macrophage-induced inflammation [45] and free LRP1 induces pro-inflammatory factor synthesis, which has the function of amplifying inflammation [46]. LRP1 also promotes the antigen-presenting function of macrophages and dendritic cells [47]. All of these suggest that LDLR and LDLR-related proteins have strong immune functions in the tumor immune microenvironment. And the high expression of LDLR in immune cells can promote the enhanced function of immune cells and facilitate the anti-tumor effect of immune cells. Therefore, targeting LDLR in the microenvironment and reversing the distribution of LDLR in the tumor immune microenvironment has some therapeutic significance.

## Conclusions

Transcriptomic and proteomic analysis revealed a novel tumor stem-associated gene set involving 65 genes. Pan-cancer analysis highlighted the multi-omics characteristics of the tumor stem-associated gene set. Breast cancer patients with high stemness scores showed the worse prognoses, accompanied by dysfunction of tumor immune microenviroment, metabolic remodeling, and cell death status. Among the tumor stem-associated genes, LDLR had the potential to promote the crosstalk between tumor stemness and immune microenvironment, which further contributed to the poor prognosis through scRNA-seq, bulk RNA sequencing, and experimental validation.

### Supplementary Information

** Additional file 1: Table S1.** 65 genes associated with the stemness of breast cancer were obtained after intersection of RNA and protein sequencing data.  **Table S2.** Survival analysis for 3132 patients with breast cancer determined 41 prognosis-related genes.

** Additional file 2: Figure S1.** The transcriptome and proteome sequencing results of 65 stemness-related genes in the SP1–SP4 breast cancer cell lines.

** Additional file 3: Figure S2.** KM survival analysis of 65 stemness genes based on 3132 breast cancer data from public databases.

** Additional file 4: Figure S3.** Lasso regression analysis using 41 immune stemness genes. (A) Coefficients of lasso regression analysis. (B) Partial likelihood deviance of lasso regression analysis.

** Additional file 5: Figure S4.** Construct survival models based on training sets. (A) The relationship between survival time and risk scores in the training set. (B) Two groups in the training set based on risk scores: high risk and low risk. (C) Expression of 9 immune stemness genes of high and low risk groups in the training set. (D) KM survival analysis of high and low risk groups in the training set.

** Additional file 6: Figure S5.** Validation of the survival model by internal validation set 1. (A) The relationship between survival time and risk scores in internal validation set 1. (B) Two groups in internal validation set 1 based on risk scores: high risk and low risk. (C) Expression of 9 immune stemness genes of high and low risk groups in internal validation set 1. (D) KM survival analysis of high and low risk groups in internal validation set 1.

** Additional file 7: Figure S6.** Validation of the survival model by internal validation set 2. (A) The relationship between survival time and risk scores in internal validation set 2. (B) Two groups in internal validation set 2 based on risk scores: high risk and low risk. (C) Expression of 9 immune stemness genes of high and low risk groups in internal validation set 2. (D) KM survival analysis of high and low risk groups in internal validation set 2.

** Additional file 8: Figure S7.** Validation of the survival model by external validation set 1. (A) The relationship between survival time and risk scores in external validation set 1. (B) Two groups in external validation set 1 based on risk scores: high risk and low risk. (C) Expression of 9 immune stemness genes of high and low risk groups in external validation set 1. (D) KM survival analysis of high and low risk groups in external validation set 1.

** Additional file 9: Figure S8.** Validation of the survival model by external validation set 2. (A) The relationship between survival time and risk scores in external validation set 2. (B) Two groups in external validation set 2 based on risk scores: high risk and low risk. (C) Expression of 9 immune stemness genes of high and low risk groups in external validation set 2. (D) KM survival analysis of high and low risk groups in external validation set 2.

** Additional file 10: Figure S9.** Effect of LDLR on survival time in pan-cancer and breast cancer.

** Additional file 11: Figure S10.** Relationship between LDLR and clinical features of breast cancer.

** Additional file 12: Figure S11.** The regulatory potential of LDLR on breast cancer stemness and proliferation. (A) Quantitative analysis of sphere formation experiments following LDLR overexpression, including both number and size. (B) Quantitative analysis of sphere formation experiments following LDLR knockdown, including both number and size. (C) Quantitative analysis of breast cancer clonogenic assay following LDLR overexpression. Relative cell viability was normalized to control (EV) cells. EV: empty vector. (D) Quantitative analysis of breast cancer clonogenic assay following LDLR knockdown. Relative cell viability was normalized to control (shNC) cell. *p < 0.05; **p < 0.01; ***p < 0.001.

** Additional file 13: Figure S12.** The correlation between immune cell infiltration and LDLR in pan-cancer.

** Additional file 14: Figure S13.** Quality control of scRNA-seq. (A) Data evaluation of scRNA-seq prior to quality control. (B) Data distribution of each sample in scRNA-seq data. (C) Data evaluation of scRNA-seq after quality control.

** Additional file 15: Figure S14.** Pre-analytical processing of scRNA-seq data. (A) Data normalization of scRNA-seq data. (B) Data distribution of each sample of scRNA-seq data is based on PCA. (C) Standard Deviation of scRNA-seq. (D) 18 cluster cell subgroups based on the SingleR algorithm. (E) Cell cycle classification based on the Tricycle algorithm.

** Additional file 16: Figure 15.** ScRNA-seq classification tree based on SingleR algorithm.

## Data Availability

The datasets analyzed in this work may be found in the Supplementary Materials or contact with the first author.
