# Supplementary figures and images for: Low-density lipoprotein receptor promotes crosstalk between cell stemness and tumor immune microenvironment in breast cancer: a large data-based multi-omics study
Source: J Transl Med. 2023 Nov 30;21:871. doi: 10.1186/s12967-023-04699-y (PMC10691045; doi:10.1186/s12967-023-04699-y)

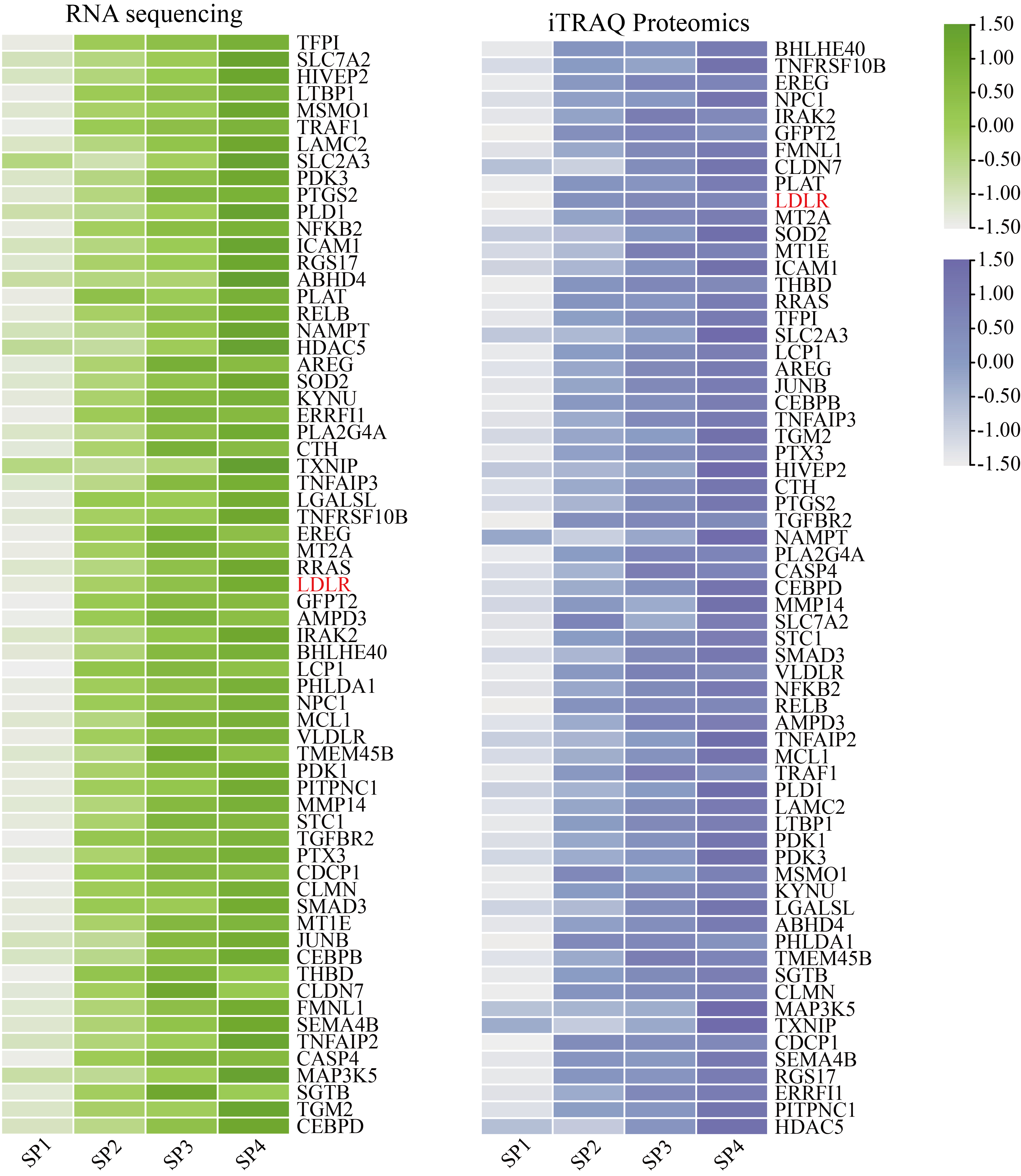

Supplement: Supplementary file 2 — Additional file 2: Figure S1. The transcriptome and proteome sequencing results of 65 stemness-related genes in the SP1–SP4 breast cancer cell lines. [file 12967_2023_4699_MOESM2_ESM.jpg]

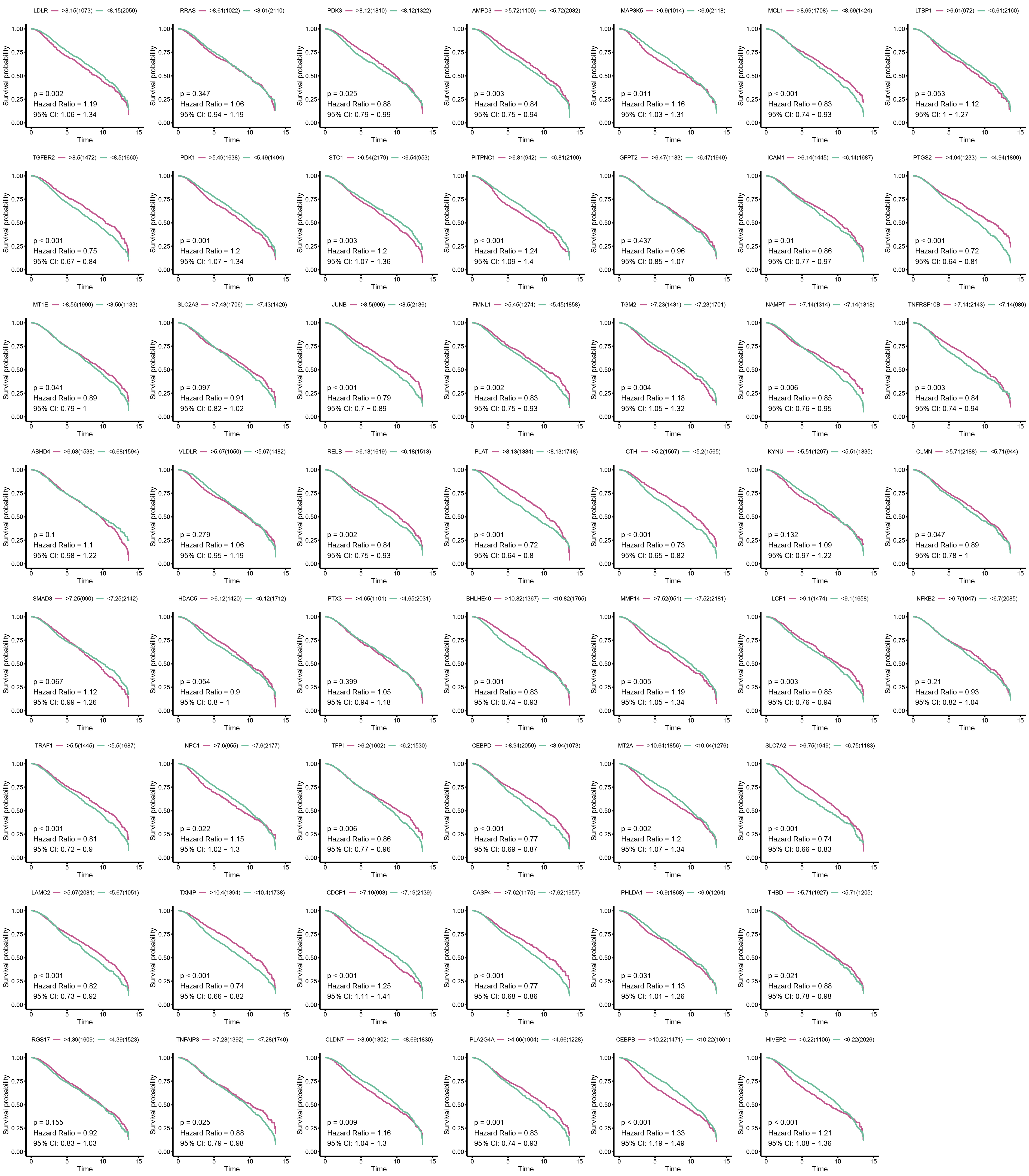

Supplement: Supplementary file 3 — Additional file 3: Figure S2. KM survival analysis of 65 stemness genes based on 3132 breast cancer data from public databases. [file 12967_2023_4699_MOESM3_ESM.jpg]

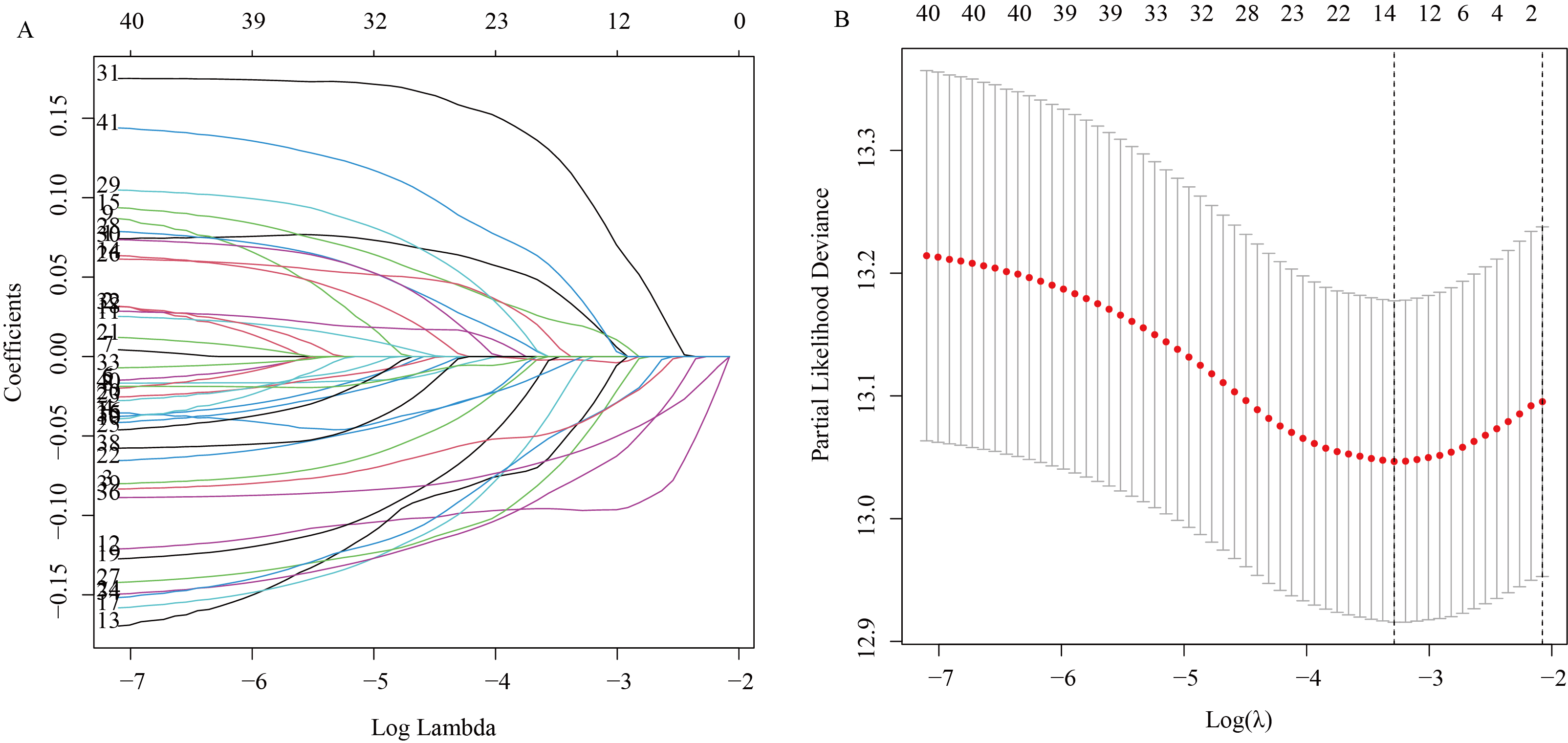

Supplement: Supplementary file 4 — Additional file 4: Figure S3. Lasso regression analysis using 41 immune stemness genes. (A) Coefficients of lasso regression analysis. (B) Partial likelihood deviance of lasso regression analysis. [file 12967_2023_4699_MOESM4_ESM.jpg]

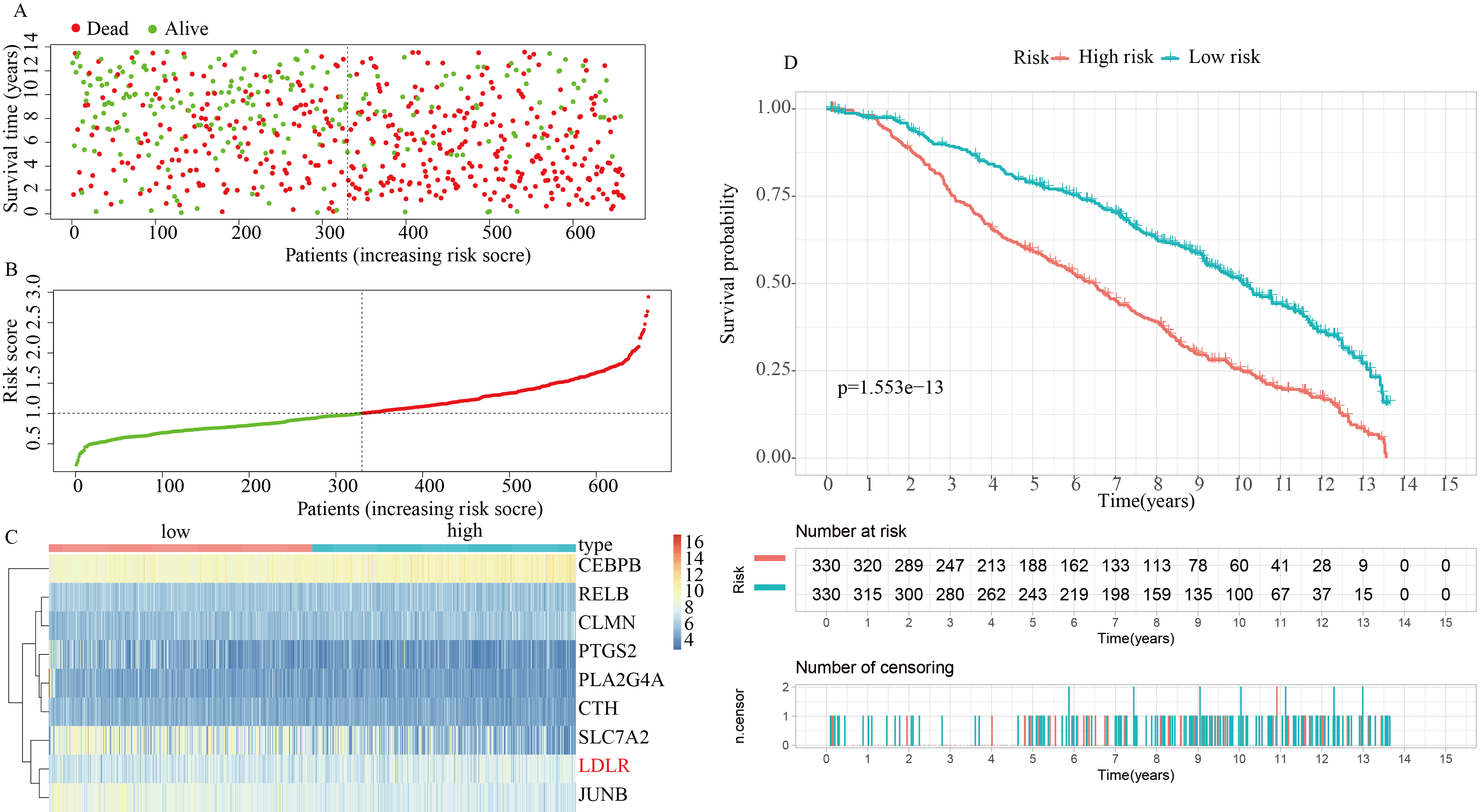

Supplement: Supplementary file 5 — Additional file 5: Figure S4. Construct survival models based on training sets. (A) The relationship between survival time and risk scores in the training set. (B) Two groups in the training set based on risk scores: high risk and low risk. (C) Expression of 9 immune stemness genes of high and low risk groups in the training set. (D) KM survival analysis of high and low risk groups in the training set. [file 12967_2023_4699_MOESM5_ESM.jpg]

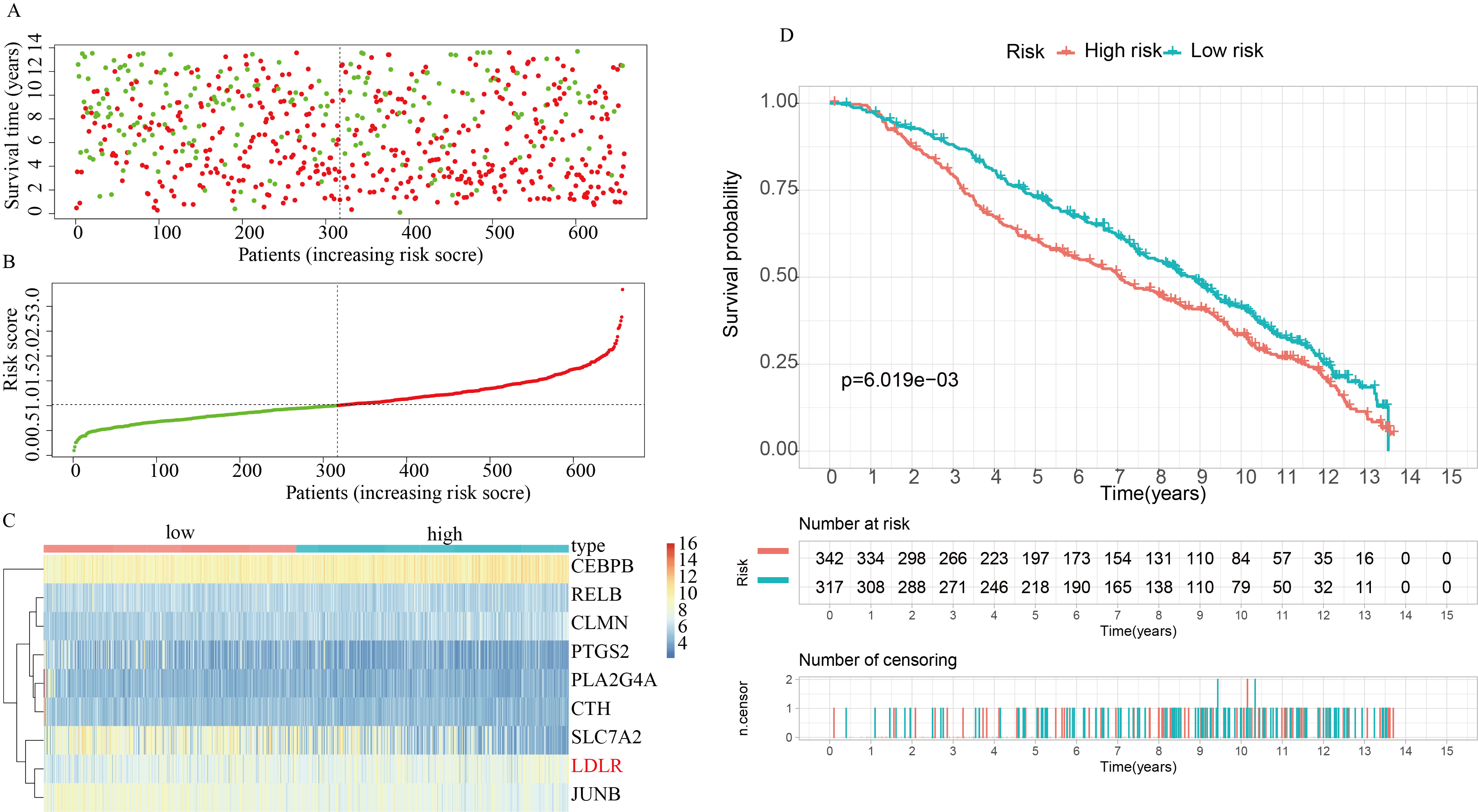

Supplement: Supplementary file 6 — Additional file 6: Figure S5. Validation of the survival model by internal validation set 1. (A) The relationship between survival time and risk scores in internal validation set 1. (B) Two groups in internal validation set 1 based on risk scores: high risk and low risk. (C) Expression of 9 immune stemness genes of high and low risk groups in internal validation set 1. (D) KM survival analysis of high and low risk groups in internal validation set 1. [file 12967_2023_4699_MOESM6_ESM.jpg]

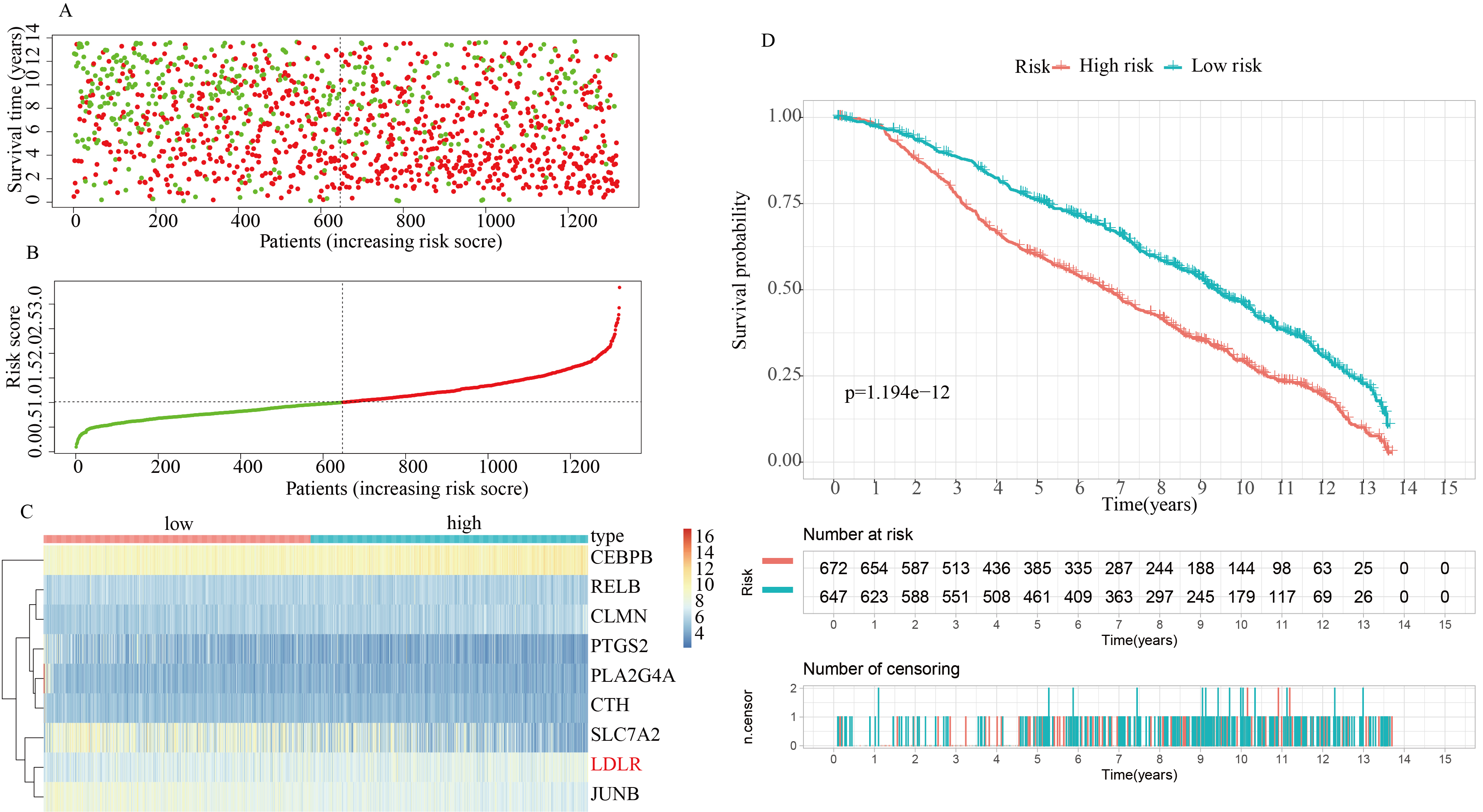

Supplement: Supplementary file 7 — Additional file 7: Figure S6. Validation of the survival model by internal validation set 2. (A) The relationship between survival time and risk scores in internal validation set 2. (B) Two groups in internal validation set 2 based on risk scores: high risk and low risk. (C) Expression of 9 immune stemness genes of high and low risk groups in internal validation set 2. (D) KM survival analysis of high and low risk groups in internal validation set 2. [file 12967_2023_4699_MOESM7_ESM.jpg]

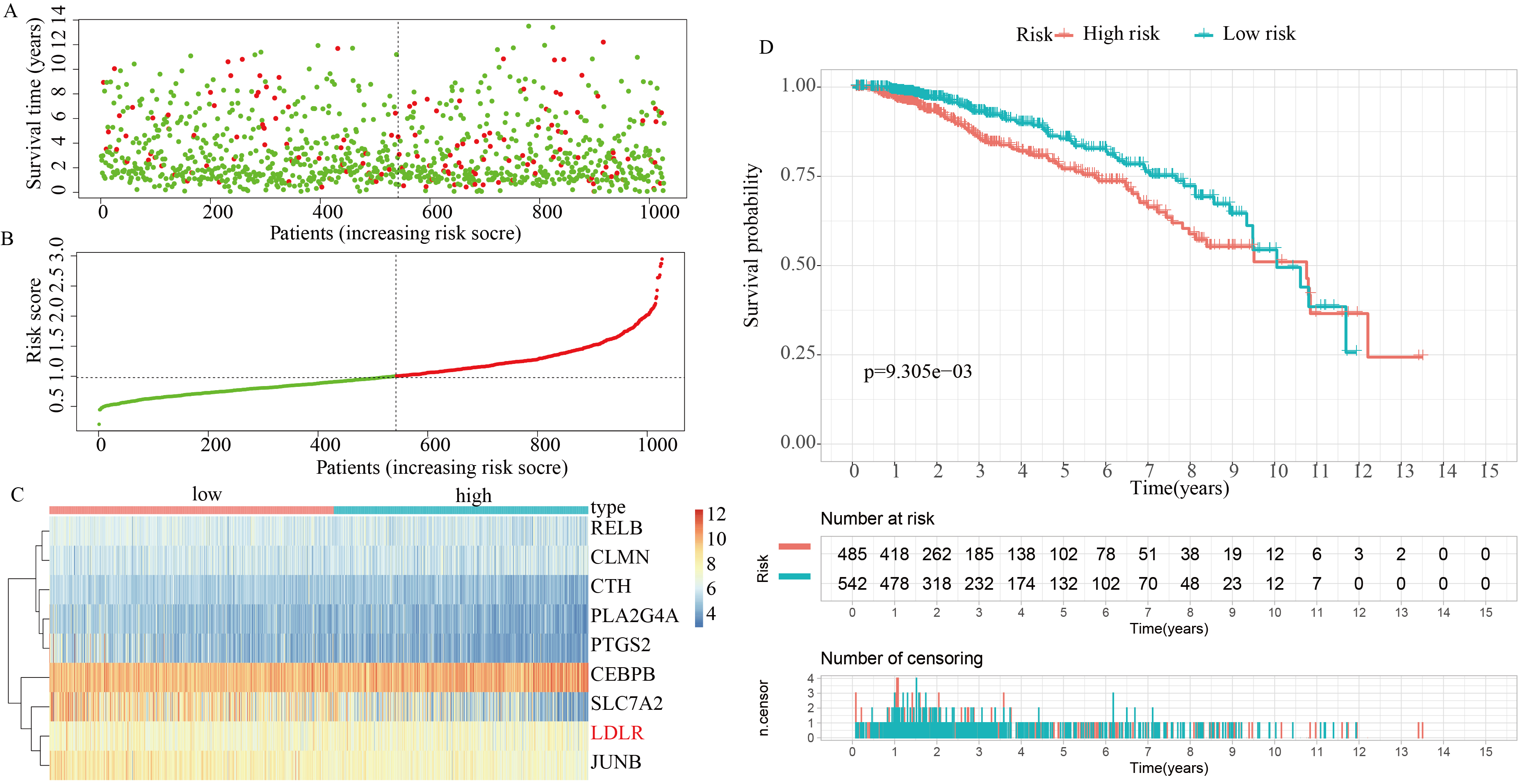

Supplement: Supplementary file 8 — Additional file 8: Figure S7. Validation of the survival model by external validation set 1. (A) The relationship between survival time and risk scores in external validation set 1. (B) Two groups in external validation set 1 based on risk scores: high risk and low risk. (C) Expression of 9 immune stemness genes of high and low risk groups in external validation set 1. (D) KM survival analysis of high and low risk groups in external validation set 1. [file 12967_2023_4699_MOESM8_ESM.jpg]

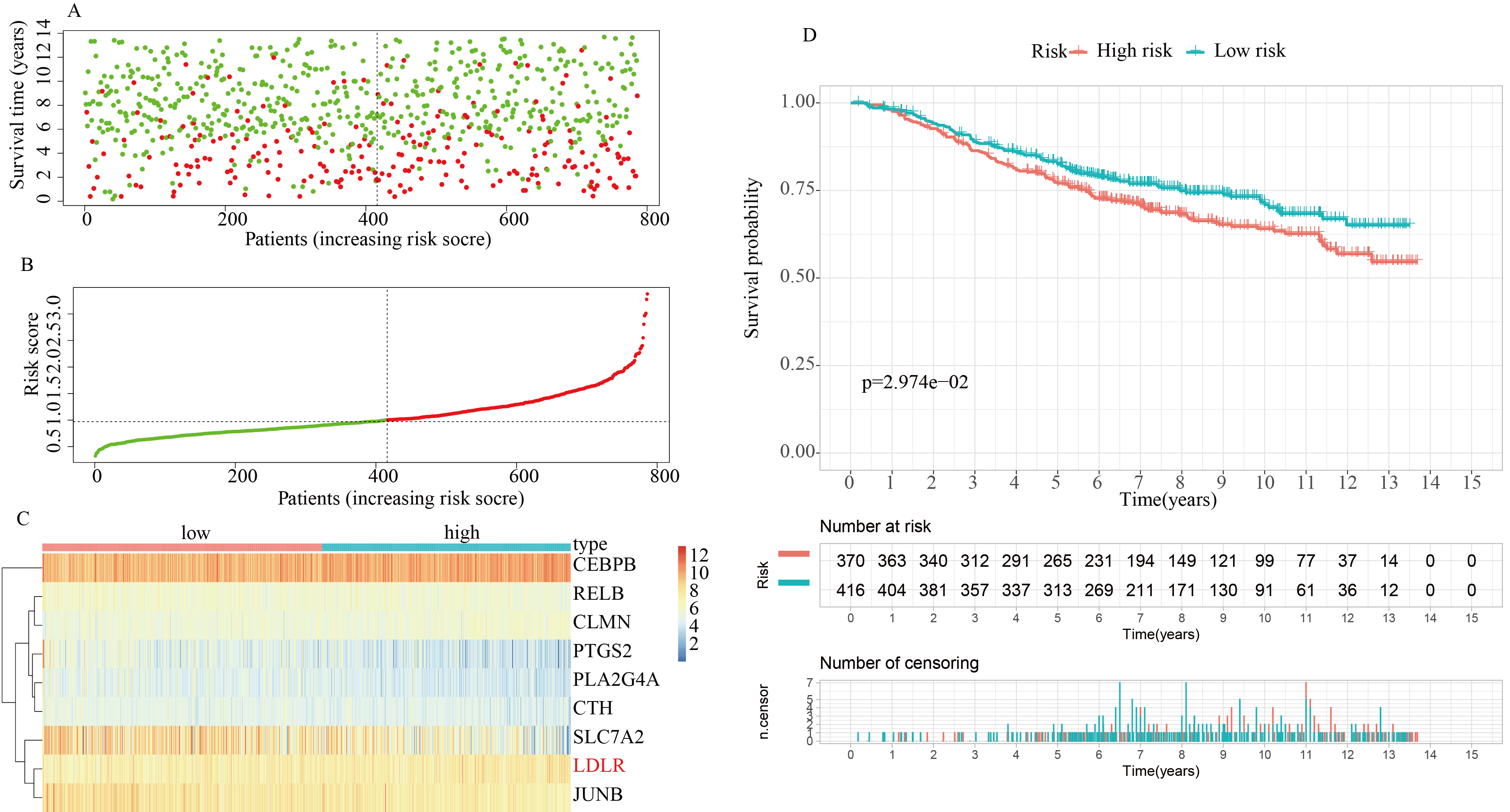

Supplement: Supplementary file 9 — Additional file 9: Figure S8. Validation of the survival model by external validation set 2. (A) The relationship between survival time and risk scores in external validation set 2. (B) Two groups in external validation set 2 based on risk scores: high risk and low risk. (C) Expression of 9 immune stemness genes of high and low risk groups in external validation set 2. (D) KM survival analysis of high and low risk groups in external validation set 2. [file 12967_2023_4699_MOESM9_ESM.jpg]

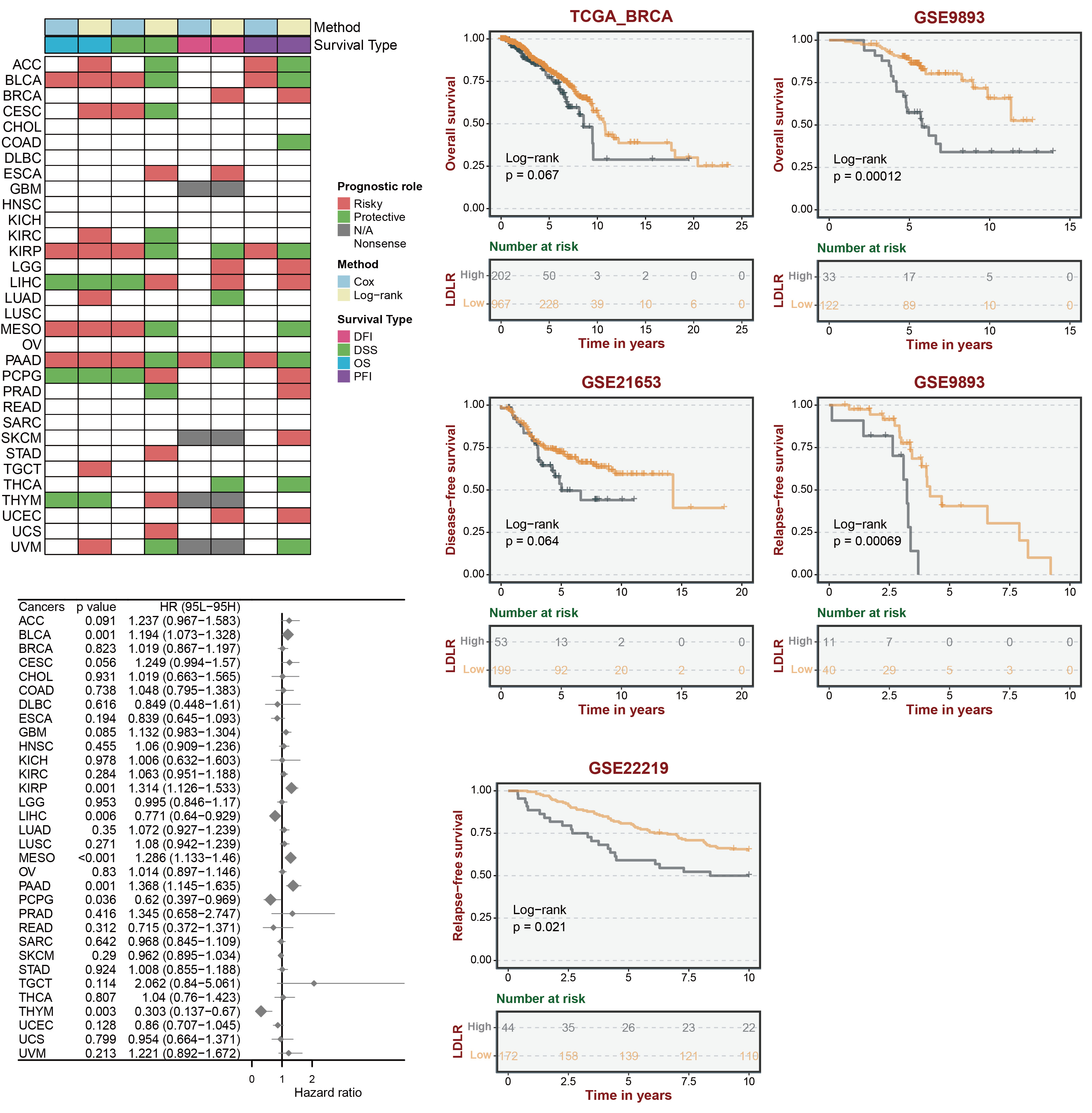

Supplement: Supplementary file 10 — Additional file 10: Figure S9. Effect of LDLR on survival time in pan-cancer and breast cancer. [file 12967_2023_4699_MOESM10_ESM.jpg]

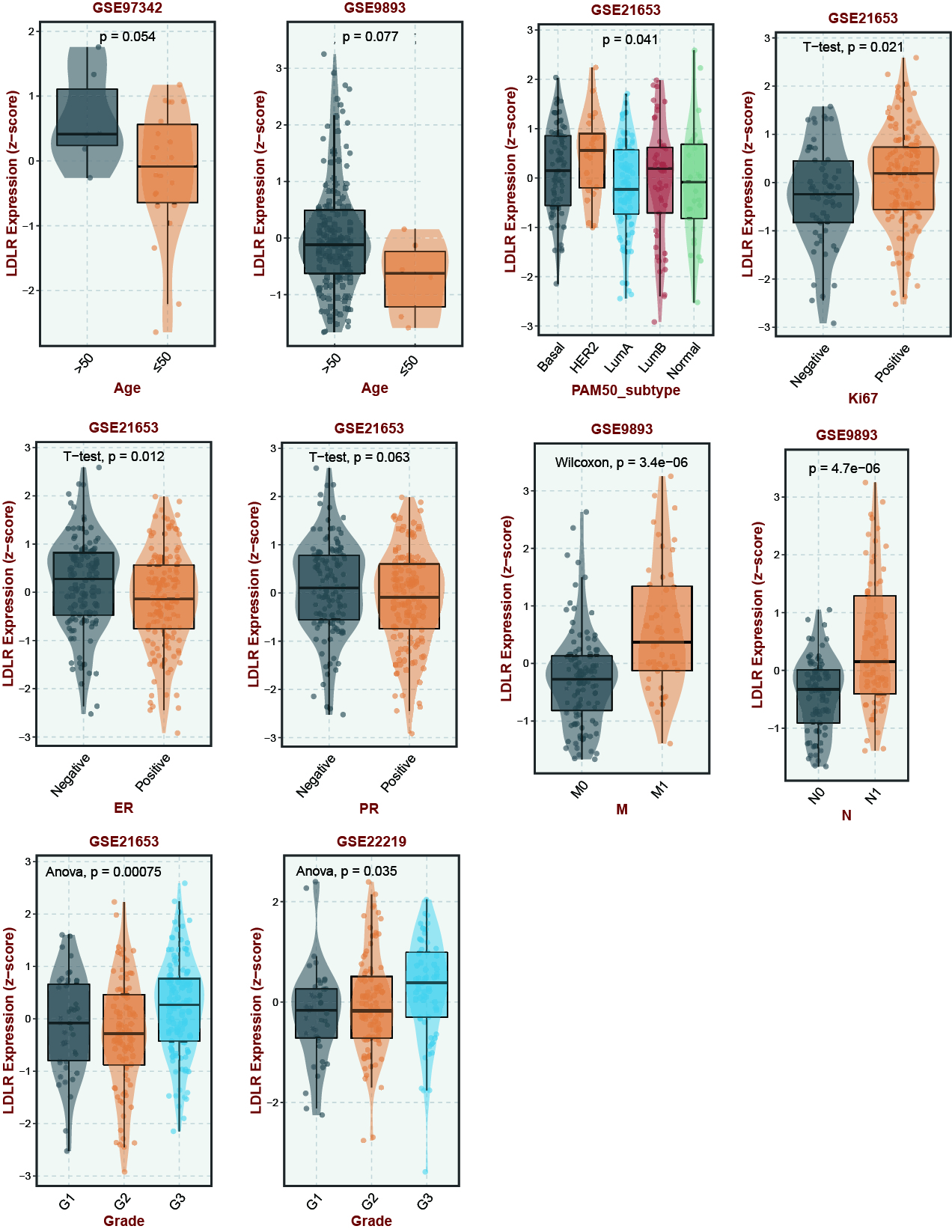

Supplement: Supplementary file 11 — Additional file 11: Figure S10. Relationship between LDLR and clinical features of breast cancer. [file 12967_2023_4699_MOESM11_ESM.jpg]

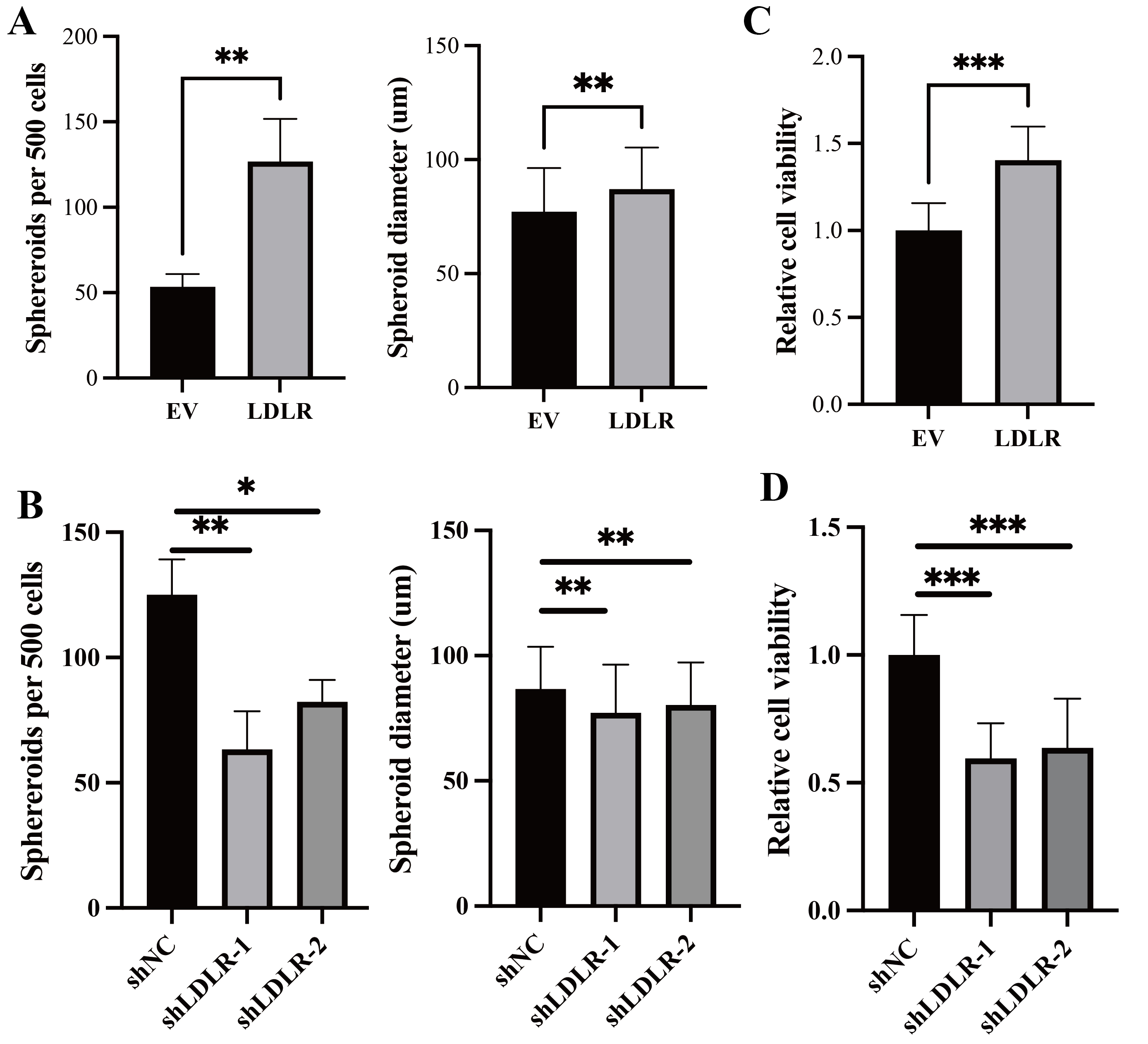

Supplement: Supplementary file 12 — Additional file 12: Figure S11. The regulatory potential of LDLR on breast cancer stemness and proliferation. (A) Quantitative analysis of sphere formation experiments following LDLR overexpression, including both number and size. (B) Quantitative analysis of sphere formation experiments following LDLR knockdown, including both number and size. (C) Quantitative analysis of breast cancer clonogenic assay following LDLR overexpression. Relative cell viability was normalized to control (EV) cells. EV: empty vector. (D) Quantitative analysis of breast cancer clonogenic assay following LDLR knockdown. Relative cell viability was normalized to control (shNC) cell. *p < 0.05; **p < 0.01; ***p < 0.001. [file 12967_2023_4699_MOESM12_ESM.jpg]

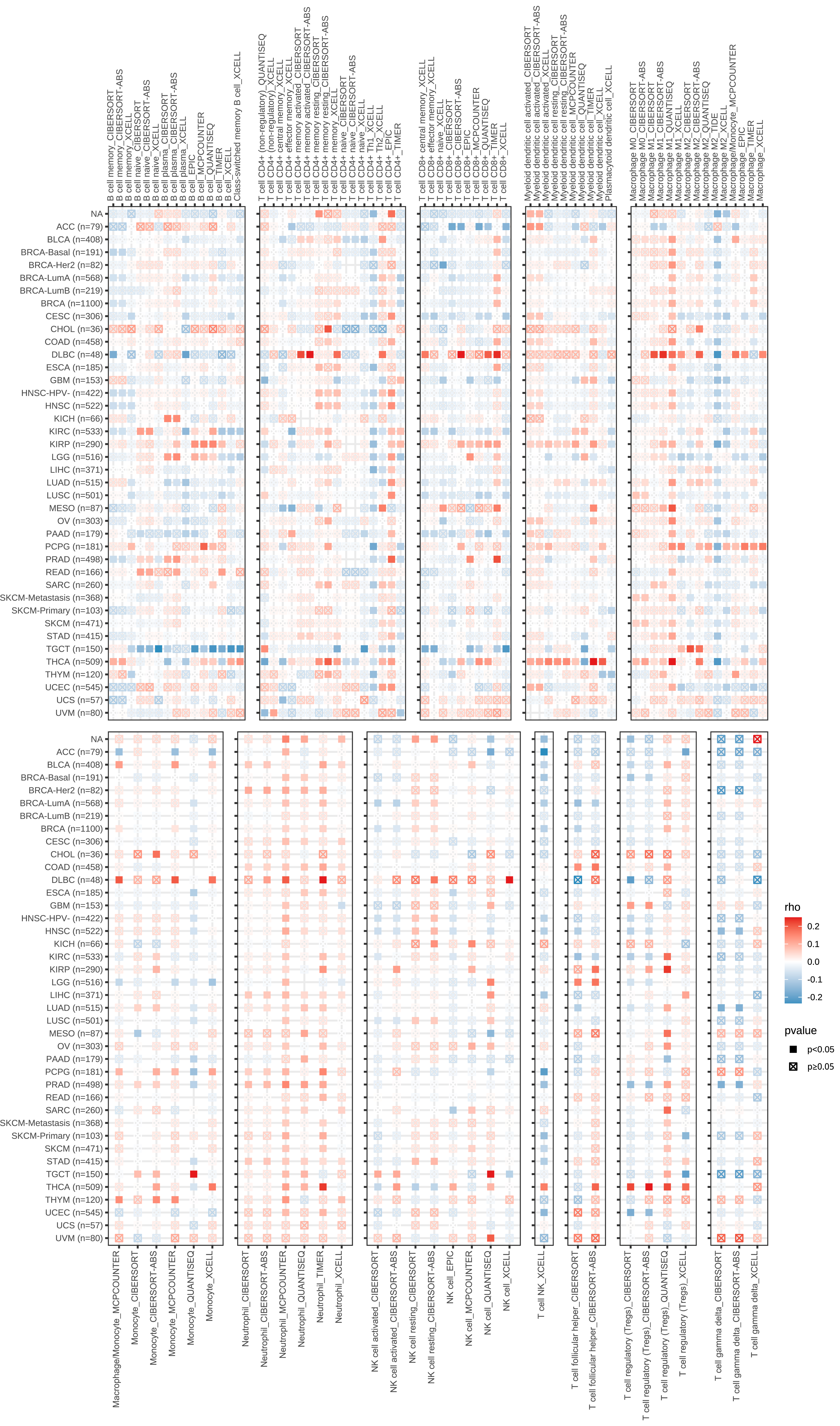

Supplement: Supplementary file 13 — Additional file 13: Figure S12. The correlation between immune cell infiltration and LDLR in pan-cancer. [file 12967_2023_4699_MOESM13_ESM.jpg]

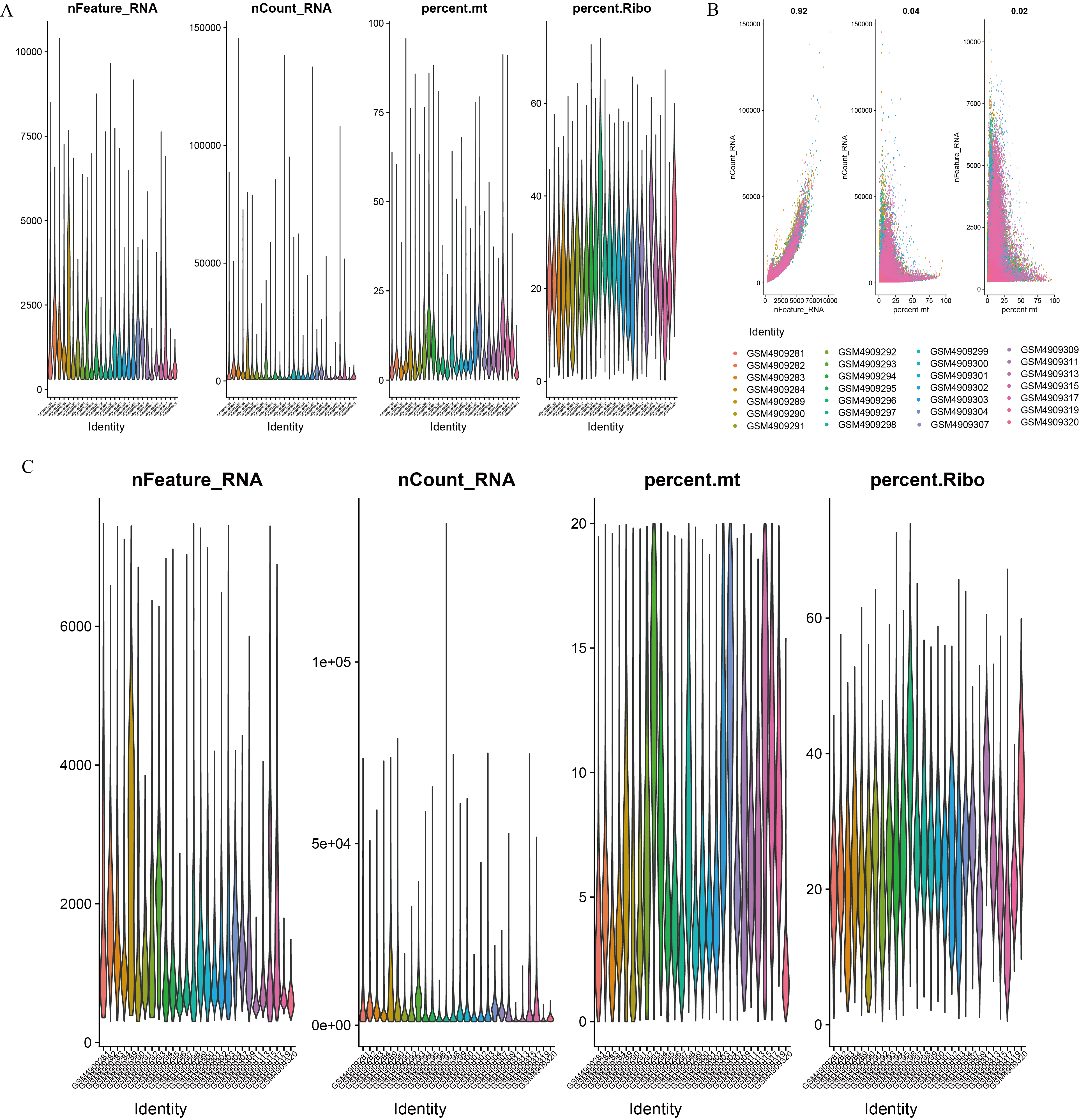

Supplement: Supplementary file 14 — Additional file 14: Figure S13. Quality control of scRNA-seq. (A) Data evaluation of scRNA-seq prior to quality control. (B) Data distribution of each sample in scRNA-seq data. (C) Data evaluation of scRNA-seq after quality control. [file 12967_2023_4699_MOESM14_ESM.jpg]

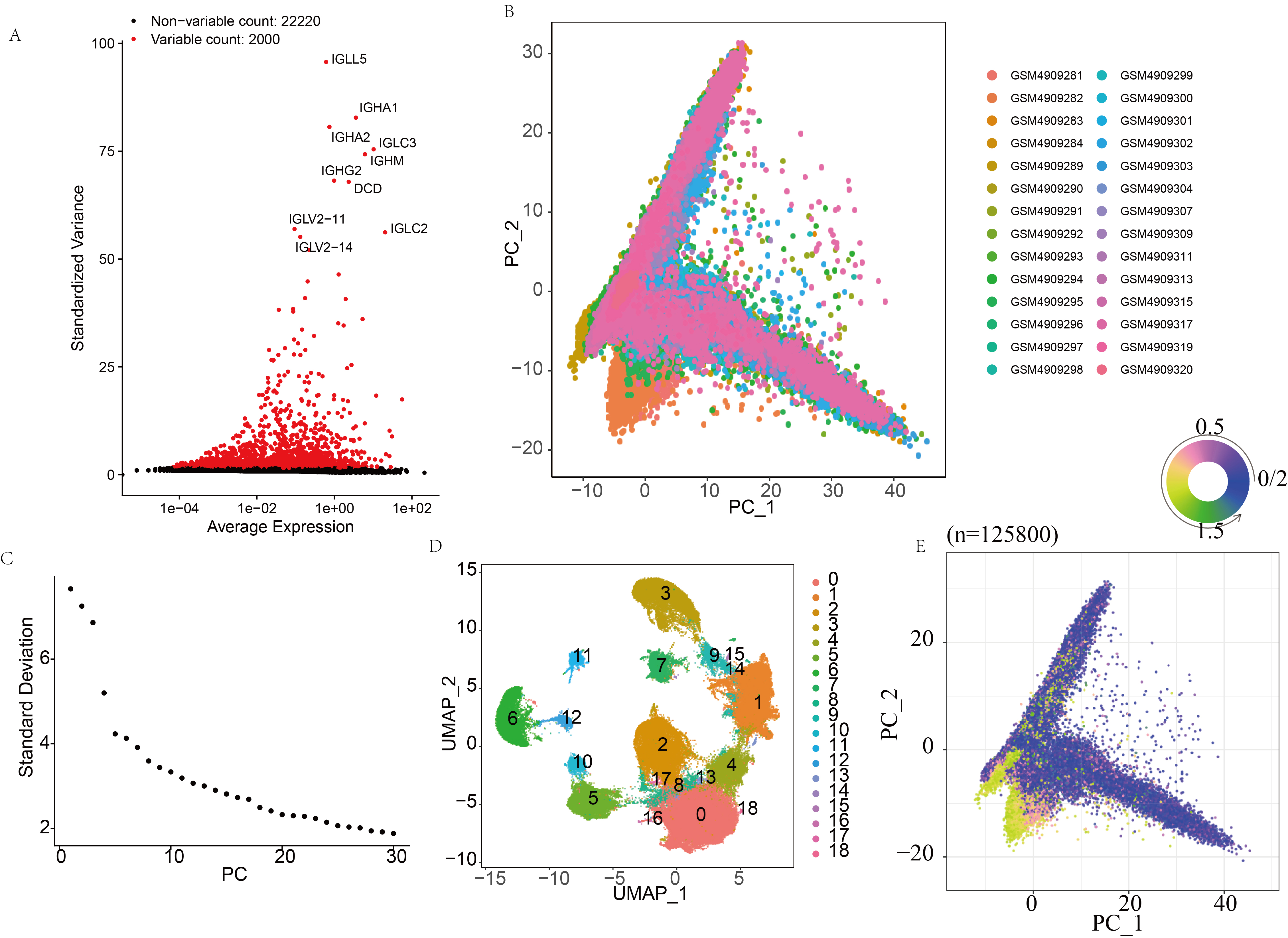

Supplement: Supplementary file 15 — Additional file 15: Figure S14. Pre-analytical processing of scRNA-seq data. (A) Data normalization of scRNA-seq data. (B) Data distribution of each sample of scRNA-seq data is based on PCA. (C) Standard Deviation of scRNA-seq. (D) 18 cluster cell subgroups based on the SingleR algorithm. (E) Cell cycle classification based on the Tricycle algorithm. [file 12967_2023_4699_MOESM15_ESM.jpg]

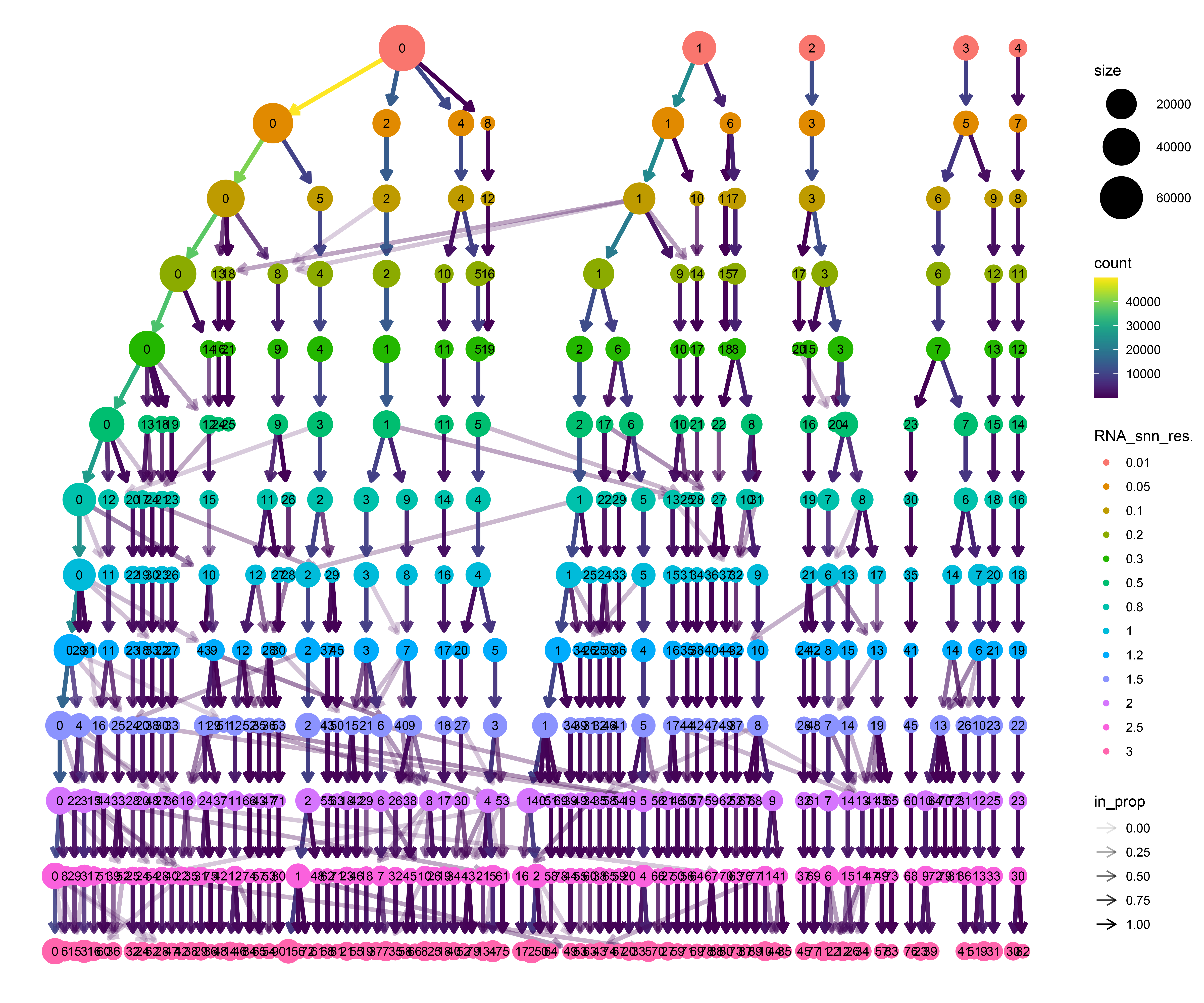

Supplement: Supplementary file 16 — Additional file 16: Figure 15. ScRNA-seq classification tree based on SingleR algorithm. [file 12967_2023_4699_MOESM16_ESM.jpg]
